# The KUPNetViz: a biological network viewer for multiple -omics datasets in kidney diseases

**DOI:** 10.1186/1471-2105-14-235

**Published:** 2013-07-24

**Authors:** Panagiotis Moulos, Julie Klein, Simon Jupp, Robert Stevens, Jean-Loup Bascands, Joost P Schanstra

**Affiliations:** 1 Institut National de la Santé et de la Recherche Médicale (INSERM), U1048, Institute of Cardiovascular and Metabolic Disease, 1 avenue Jean Poulhès, 31432 Toulouse, France; 2 Université Toulouse III Paul-Sabatier, 118 route de Narbonne, 31062 Toulouse, France; 3 School of Computer Science, University of Manchester, Kilburn Building, Oxford Road, Manchester M13 9PL United Kingdom

## Abstract

**Background:**

Constant technological advances have allowed scientists in biology to migrate from conventional single-omics to multi-omics experimental approaches, challenging bioinformatics to bridge this multi-tiered information. Ongoing research in renal biology is no exception. The results of large-scale and/or high throughput experiments, presenting a wealth of information on kidney disease are scattered across the web. To tackle this problem, we recently presented the KUPKB, a multi-omics data repository for renal diseases.

**Results:**

In this article, we describe KUPNetViz, a biological graph exploration tool allowing the exploration of KUPKB data through the visualization of biomolecule interactions. KUPNetViz enables the integration of multi-layered experimental data over different species, renal locations and renal diseases to protein-protein interaction networks and allows association with biological functions, biochemical pathways and other functional elements such as miRNAs. KUPNetViz focuses on the simplicity of its usage and the clarity of resulting networks by reducing and/or automating advanced functionalities present in other biological network visualization packages. In addition, it allows the extrapolation of biomolecule interactions across different species, leading to the formulations of new plausible hypotheses, adequate experiment design and to the suggestion of novel biological mechanisms. We demonstrate the value of KUPNetViz by two usage examples: the integration of calreticulin as a key player in a larger interaction network in renal graft rejection and the novel observation of the strong association of interleukin-6 with polycystic kidney disease.

**Conclusions:**

The KUPNetViz is an interactive and flexible biological network visualization and exploration tool. It provides renal biologists with biological network snapshots of the complex integrated data of the KUPKB allowing the formulation of new hypotheses in a user friendly manner.

## Background

During the past decade major advances in biological research, mainly in the field of high throughput analysis (e.g. –omics), has led to an exponential increase in available experimental data, produced through a variety of techniques including DNA, miRNA [1] and antibody arrays [2], next generation sequencing technologies [3] and mass spectrometry [4]. This switch in life sciences towards multi-omics approaches has created a gap in the provision of bioinformatics tools capable of combining this data. More importantly, there appears to be a shortage in efficient tools that would aid the bench biologist to i) simplify and categorize the results of a multi-omics approach which usually come into the form of long lists ii) visualize different information layers which can be mined from multi-omics approaches and aggregate useful pieces into biochemical pathways and/or biological function groups iii) combine steps (i) and (ii) in a repeatable and reusable fashion so as to extract meaningful outcomes regarding the biological system under investigation and iv) combine all the aforementioned steps to formulate plausible hypotheses, possibly applied to similar systems (e.g. systems functioning in the same tissue/organ/similar disease situation) and eventually design new experiments for hypothesis validation.

The renal biology field is facing similar problems. Although kidney diseases have been extensively studied in different species (i.e. human, mouse, rat), this wealth of information remains hidden across several layers of public data and/or literature repositories. Although considerable effort has been devoted in aggregating data from several resources [5-7], the resulting databases fail to meet the multi-omics attribute and remain dispersed across the web. To address this problem, we developed the Kidney and Urinary Pathway Knowledge Base (KUPKB) [8], a publicly available repository which organizes an important amount of existing knowledge regarding renal tissue, cell and disease categorization, using Semantic Web technologies [9]. The KUPKB can be queried through the user-friendly iKUP browser, [8], accessible at http://www.kupkb.org.

The iKUP comprises a powerful tool in terms of speed, selectivity and descriptive power. Nevertheless, its nature restricts the user to viewing the query results in tabular format, only skimming the surface of the rich interconnected data otherwise available in the KUPKB. In addition, although it succeeds in displaying findings hidden in scattered repositories, such as the expression of a set of genes under a very specific combination of kidney tissue, cellular type and disease, it remains unable to map this information to interaction networks available as background information and in a multiple species manner.

The value of biological network representations has been extensively analyzed in bioinformatics and systems biology literature [10,11]. Some important aspects include the ability to capture fixed snapshots of cellular states [12] otherwise hidden in tables, to infer functional associations [13], to reduce complexity by combining protein interaction, gene expression and metabolic profiles in a single image [14], and perform pattern recognition in a network snapshot [15].

In this article, we describe KUPNetViz, an interactive biological network querying and visualization application. Its main purpose is to assist renal scientists to extend their research by providing an alternative KUPKB data image depicting interactions among the queried molecules and their neighbors, coupled with functional and biochemical pathway annotation in both a species dependent and independent manner. The main tasks supported and promoted by KUPNetViz are: i) the display, exploration and manipulation of general protein-protein interaction as well as functional and biochemical pathway association networks for a number of widely studied mammalian species, ii) the transformation of these general networks to kidney specific networks through their association with related gene/protein/miRNA expression datasets, iii) the mining of possibly hidden relationships among co-regulated and/or directly interacting (“neighboring”) entities under several combinations of kidney anatomies and/or disease models and iv) the extrapolation of both expression data and network interactions across different diseases, anatomies but also different species facilitating the quick formulation and screening of biological hypotheses and the design of new experiments.

The molecule and interaction visual representations in KUPNetViz have been inspired by previous work in the field [16] with several additions and significant modifications to fit for use. To demonstrate its value and necessity in kidney research studies and its ability to complete the aforementioned tasks, we present two usage examples that exemplify that the KUPNetViz clearly extends the functionalities of the iKUP. Specifically, we found substantial additional evidence for a role of calreticulin in renal disease in humans [8] by showing that calreticulin is involved in a larger interaction network in renal graft rejection. In addition, we propose a novel association of the inflammatory axis interleukin-6 (IL6)/IL6 receptor with the progression of polycystic kidney disease.

## Implementation and functionality

### General architecture

The KUPNetViz is a web application developed using PHP for server side programming and jQuery ( http://www.jquery.org) coupled with static HTML for the client side. The backend of the application is a MySQL database hosting the background knowledge used to build the mappings among entities, the network interactions, annotation data and properly parsed experimental data that make up the most important part of the KUPKB. The backend database can be very easily updated and maintained as it can be rebuilt in an automated fashion with a single one-line command, through a series of Perl scripts that download, parse and import KUPKB data and the background knowledge resources into the schema, using a wrapper script and a simple YAML ( http://www.yaml.org) configuration file. The resulting interaction networks from user queries along with experimental data mappings (gene, protein, miRNA expression and statistical significance) are rendered, visualized and controlled using the Cytoscape Web graph visualization library [17].

### Background knowledge

The background knowledge integrated in KUPNetViz from KUPKB has been extensively described elsewhere [8,18]. Briefly, gene, protein and miRNA annotations are derived from NCBI gene, UniProt [19] and Ensembl [20], and Microcosm [21] respectively. For the mappings among the various biological entities and accession numbers, we used the mapping files provided by NCBI (ftp.ncbi.nlm.nih.gov) as well as the Biomart web services [22]. Files provided by NCBI were also used for the mapping of genes to their respective GO terms. Molecule interactions are extracted from the STRING protein-protein interaction database [23] by parsing publicly available flat files and miRNA to gene interactions are extracted from the Microcosm miRNA target files ( http://www.ebi.ac.uk/enright-srv/microcosm/htdocs/targets/v5/). Biochemical pathway information was derived from KEGG [24] using its freely available web service API to download and construct mappings between genes and their respective pathways.

### Experimental data

The experimental data integrated in KUPNetViz and used to map gene/protein/miRNA abundance to the biochemical networks constructed by querying the application has been extensively described elsewhere [8]. Briefly, the knowledge base currently contains over 220 experiments spanning several biological layers (gene, protein, miRNA and metabolite abundances) derived from published articles and public repositories (e.g. GEO). In some cases data are reported as extracted from the respective publication (coupled with statistical significance where available) while in other cases data had to be reanalyzed (mostly microarray data) before integration. All experimental data are derived from studies related to several kidney diseases and anatomies and are manually curated.

### Usage and navigation

**Figure 1:**
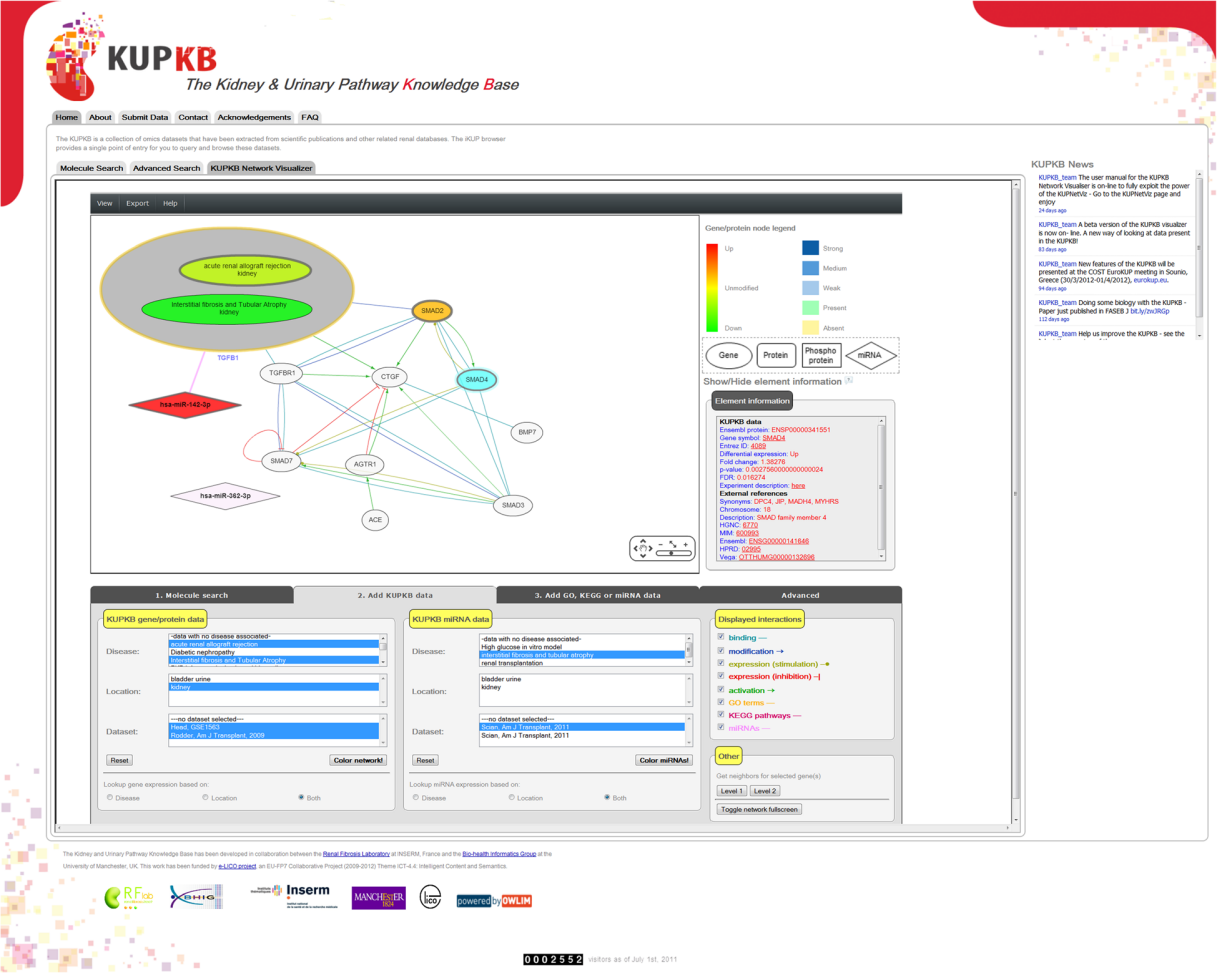
**A snaphsot of KUPNetViz in action.** Under the network view, the KUPKB data mapping tab is displayed, where the researcher can map onto the network several gene/protein/miRNA expression datasets and apply different criteria and combinations of kidney location(s), disease(s) and data from published studies. This snapshot focuses on the down-regulation of TGFβ1 in kidney in two renal disease states, namely in acute renal allograft rejection and interstitial fibrosis and tubular atrophy, and the up-regulation of the miRNA that targets TGFβ1 in interstitial fibrosis and tubular atrophy, as derived from two published studies.

The KUPNetViz application consists of a single web page divided in three main parts: i) the interaction network canvas, ii) the annotation section with legends regarding the network components and a dynamic subsection which displays information and external links for the selected network elements, and iii) the application controls which are organized in four tabs containing data search and mapping options as well as more advanced application controls. This configuration allows the user to easily navigate through the application and explore and map KUPKB data without leaving the main network view. A snapshot of the application is presented in Figure 1. The usage of the application is self-guiding and can be summarized in three basic steps: i) search using a molecule list of interest, querying one or more of the supported species, ii) mapping of experimental data to the network, by navigating and selecting through multiple renal locations and diseases associated with the queried molecules and iii) association with biological functions, processes and cellular components, with biochemical pathways and with miRNAs targeting the entities of interest.

### Data input

The user can query KUPNetViz using a variety of molecule identification types, including HUGO gene symbols (e.g. “CALR”, but the search is case-insensitive), Entrez accession numbers (e.g. 811), UniProt accessions (e.g. P27797), Ensembl gene and protein IDs (e.g. ENSG00000179218 or ENSP00000320866), miRBase accessions (e.g. hsa-miR-362-3p) or by simple free text (e.g. “calreticulin”). All the above accessions and search terms are equivalent to *calreticulin* and can be used in mixed identification types. Four different mammalian species are currently supported, namely human, mouse, rat and dog. A strong feature of KUPNetViz, which is rare among other application of the same kind, is the ability to query multiple species for protein-protein network reconstruction at the same time.

### Extrapolation of protein–protein interactions across species

The extrapolation procedure followed by KUPNetViz is quite simplistic and is performed by assuming that a protein-protein interaction which has been recorded experimentally or predicted in e.g. humans but not in mice has a probability of being also present in mice (or any other of the supported by the application mammalian species). Thus, instead of aligning networks from different organisms (in similar taxonomies) the tool creates a meta-network with “super-nodes” and “super-edges” where protein-protein interactions are shared among species. Although this assumption involves certain risk regarding its biological validity, it allows the quick creation of hypotheses regarding those protein-protein interactions and the quick screening from the experimentalist. The extrapolation assumption to super-nodes and super-edges is also based on a certain “bias” in the number of biological studies regarding each organism, as there is a normal and understandable trend towards the study of human biomaterial (cell lines, healthy or diseased tissues) as compared to other organisms. For example, a simple search of the term “homo sapiens” in Pubmed yields a number of studies which is one order of magnitude greater (12547599) than the respective number of studies for “mus musculus” (1159658) at the time of search. This is reflected also in the number of protein-protein interactions: using a calreticulin-centered first level network (the first neighbors of calreticulin) for human, 365 protein-protein interactions are found (interaction score threshold: 0.2) while the same number for mouse is 8 (same threshold).

### Running time

The running time of the KUPNetViz depends on two factors: i) the performance of the network layout algorithm and the visualization itself (e.g. supported network size) which depends on the Cytoscape Web library and ii) the querying performance of the application regarding the mapping of renal –omics datasets to the network. The latter depends on the number of queried molecules. Typical queries with a few molecule names complete in a few seconds. The only operations that are quite time consuming both in terms of querying and visualization time are the search for second level neighbors of selected nodes and the case of many queried molecules when working in multi-species mode. Finally, the speed of the network layout depends on the user’s machine as the network rendering takes place in the user’s browser.

### Data export

Network export is available through the respective functions of the Cytoscape Web library. The user has the ability to export the resulting network after querying and processing, in a variety of text formats (SIF, GraphML, XGMML and Arena3D format [25]) as well as high-quality image formats (PDF, PNG and SVG). The text formats allow the import of the resulting networks into other graph analysis applications (such as Cytoscape) for further explorations and network property analysis. The supported text formats are sufficient for compliance with most current biological network visualization tools.

## Results

To demonstrate the added value of the KUPNetViz we present two case studies, revealing insights that would require significant effort to mine using the iKUP only. Specifically, we explored the role of calreticulin, a protein involved in renal disease in animals, in a larger interaction network in renal graft rejection (interstitial fibrosis and tubular atrophy) and its association with other functional and pathway elements and the association of the inflammatory axis interleukin-6 (IL6)/IL6 receptor with the progression of polycystic kidney disease.

### Case study 1: the role of calreticulin in renal graft rejection

Calreticulin is a protein involved in renal disease in animals [26]. Using the iKUP browser to explore calreticulin entries in the KUPKB, we demonstrated for the first time that calreticulin expression was induced in human renal graft rejection, an *in silico* hypothesis that was then experimentally confirmed [8]. To better understand the role of calreticulin in renal graft rejection, we sought to investigate the following questions: 1) was calreticulin acting as an independent player or as a part of a bigger protein network and 2) what were the processes associated with calreticulin dysregulation. These questions could not be answered using the iKUP browser alone, as they required knowledge about protein-protein interactions and protein annotation with biological processes.

**Figure 2:**
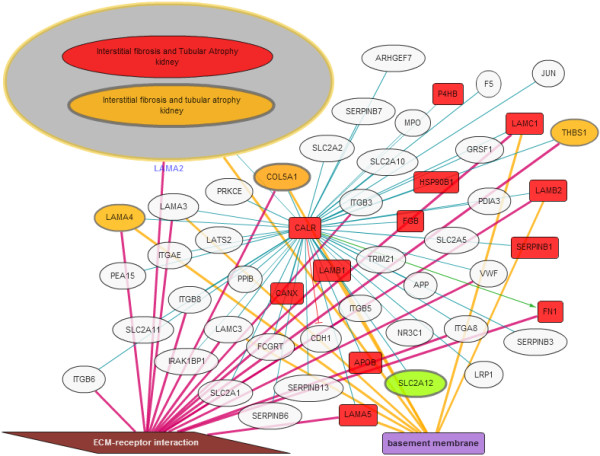
**Calreticulin network.** Analysis of the calreticulin network shows that not only calreticulin expression is modified in renal graft rejection but also many of the genes/proteins (34%) with known interactions with calreticulin. In addition specific processes/pathways are enriched including extracellular matrix interaction and basement membrane proteins. These are processes and pathways known to be involved in renal graft rejection. Overall projection of the KUPKB data on the calreticulin interaction network clearly exemplifies a role of calreticulin in renal graft rejection.

We used the KUPNetViz to search for “calreticulin” in human and visualized neighboring genes/proteins (first level neighbors, interaction threshold: 0.2). We next annotated the network with renal graft rejection data contained in the KUPKB, GO processes and KEGG pahways. To perform this procedure, we selected “interstitial fibrosis and tubular atrophy” from the kidney disease panel. This disease model was associated with, namely “Nakorchevsky, J Am Soc Nephrol, 2010”, “Rodder, Am J Transplant, 2009” and “Scherer, Nephrol Dial Transplant, 2009”. In addition to these datasets, we also selected “ECM-receptor interaction” pathway and “basement membrane” cellular component from the GO and KEGG panels respectively. The resulting network is presented in Figure 2.

Overall, we observed that calreticulin was at the centre of a network where many genes and proteins were also modified in this pathology, and that most of these biomolecules were part of the basal membrane and involved with cell to extracellular matrix interactions (ECM). Specifically, the ECM-receptor interaction pathway is mostly upregulated, as observed from the orange to red colour scale of the nodes connected to calreticulin (Figure 2).

ECM and basement membrane are known to be significantly altered in renal graft rejection [27] and the analysis performed using our tool links calreticulin to these pathological processes. Furthermore, basement membrane proteins identified as interacting with calreticulin such as laminins and fibronectin (LAMA2, LAMA4, LAMA5, LAMB1, LAMB2, LAMC1 and FN1 in Figure 2) have been identified to be upregulated in renal graft rejection [28,29]. Altogether, the use of KUPNetViz provides additional, pathway-based, evidence for calreticulin as a valuable target in renal graft rejection. Although it would not have been impossible to find these links otherwise, the use of the tool presented in this article significantly accelerated this discovery. For example, a search using Pubmed did not return any results when querying for “laminin fibronectin calreticulin”. Furthermore, querying classic network visualization tools such as the tool provided in the STRING database [23] website lack background knowledge on kidney diseases.

### Case study 2: potential involvement of IL6 and the IL6 receptor in the progression of polycystic kidney disease

IL6 is a pro-inflammatory cytokine that has been previously described as a pro-fibrotic mediator in liver [30], in lung and in skin [31] models of fibrosis. However, current evidence for a possible role of IL6 in the development of renal fibrosis is limited. The only available evidence of this link in the kidney was published recently in a study demonstrating that mice with genetic blockade of IL6 were protected against the development of renal fibrosis [32]. In addition, the authors also observed increased IL6 expression levels in kidney biopsies of chronic kidney disease patients compared to age-matched control biopsies. IL6 is a peculiar cytokine as, although it is expressed by a large variety of cells, it can only target a low number of those, due to the very limited expression of its receptor (IL6R) [33].

**Figure 3:**
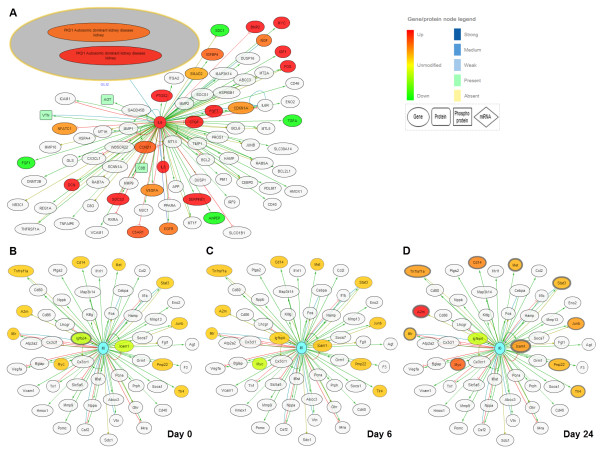
**The IL6/IL6R axis network for human and rat.** The IL6/IL6R axis expression network created by querying the KUPNetViz for IL6 and IL6R and using the first level interaction neighbors of IL6. **(A)** The network associated with IL6/IL6R is mostly up-regulated in patients with PKD as observed after mapping the results of three relevant studies in human (namely “Lai, Proteomics Clin Appl, 2008”, “Mason, Proteomics Clin Appl, 2009” and “Song, Hum Mol Genet, 2009”), **(B**-**D)** Progressive upregulation of the IL6/IL6R network in PKD in rat model as derived from the study of Koupepidou *et al.*[36]. The three figures depict three distinct stages of the disease, specifically **(A)** day 0, **(B)** day 6 and **(D)** day 24, after mapping the three experimental conditions in the dataset “Koupepidou, BMC Nephrol, 2010”.

To further investigate the possible role of IL6 and IL6R, we performed a search for these molecules in the iKUP which demonstrated that their renal expression in normal conditions seemed to be limited to the collecting duct, a tubular structure of the kidney [34]. The results also showed that IL6 and IL6R were up-regulated in both human polycystic kidney disease (PKD) and in a rat PKD model [35,36]. To our knowledge, there are few data linking IL6 to PKD but a recent study [37] demonstrated that patients with PKD had higher level of serum IL6 as compared to controls. In order to obtain further support for this link, we next searched for IL6 and IL6R in the KUPNetViz. In humans, the network associated with those two entities obtained by querying for and displaying their first level interacting neighbors was mostly up-regulated in patients with PKD (Figure 3A). This outcome was derived after mapping three KUPKB datasets related to PKD, namely “Lai, Proteomics Clin Appl, 2008”, “Mason, Proteomics Clin Appl, 2009” and “Song, Hum Mol Genet, 2009”, [35,38,39] (disease filter: “autosomic dominant polycystic kidney disease” and “PKD1 autosomic dominant kidney disease”). More importantly, in mouse and rat models of PKD, up-regulation of the network increased with the severity of the disease as levels of up-regulation increased (i.e. orange to red) and the p-values decreased with disease progression (thickening of node border). This outcome was derived after mapping the dataset “Chen, BMC Res Notes, 2008” for mouse [40], and the dataset “Koupedidou, BMC Nephrol, 2010” for rat [36]. The latter is depicted in Figure 3B-D. To conclude, by using the KUPNetViz we were able to generate a novel hypothesis, suggesting for the first time that the IL6/IL6R axis might be an important player in PKD and a valuable target in this disease. In the future, experiments will be needed to confirm or refute this hypothesis but this could represent a major step forward in understanding and handling this common and life-threatening genetic disease.

## Discussion

The wealth of biological information regarding kidney disease that can be extracted from the current version of the KUPKB is currently presented in tabular format. Given the possibly long returning lists of molecules, the nature of the knowledge base and the fact that it contains hierarchical and protein interaction data, we sought to visualize these interactions through a gene network visualization module. Prior to its development we tried to map experimental data to some of the numerous existing software packages that have been developed for biological network visualization and exploration and at the same time suited at least partially our needs (mapping of possible multi-omics data, integrated background databases, customizable data sources). These tools included EGAN, [16] and Biological Networks, [41]. In the case of EGAN, the feedback included the excessive amount of displayed information, the view complexity derived from the multiple data sources, the inability to simultaneously display expression data from multiple experiments and the moderate performance of the application regarding big networks. Another major issue with EGAN is the lack of directionality in gene-to-gene edges. On the other hand, in the case of Biological Networks, the users were overwhelmed mostly by the amount of functionalities, the complex interface and the multiple background knowledge data sources, features that might fascinate a bioinformatician but often frustrates a bench biologist seeking clear-cut specific answers instead of heavy and complex multi-functionalities. In addition, Biological Networks requires a local installation which is often a disadvantage, mostly in terms of maintenance.

The above tools comprise only a small example out of a nowadays large pool of tools allowing the combined view of protein-protein interaction networks and abundance data [14]. Leaving aside the domain-specific nature of KUPNetViz, most of the packages presented in the first table of [14] meet the criteria of combined views of interaction and abundance data (e.g. VANTED, [42], GENeVis, [43]) and are equipped with high-level functionalities (e.g. network clustering algorithms, [44] dimensionality reduction methods, [45], visualization of extremely large networks, [46], and network module detection, [47]). However, although the above tools excel in network-related functionalities and visualization quality, none of them comes with a complete and yet simple set of bundled background databases (protein-protein interaction, element annotation, functional and pathway associations) in one place, a fact which is essential for the researcher. Other solutions including Pajek, [48], and yEd ( http://www.yworks.com/en/products_yed_about.html) comprise also excellent network analysis tools but are very generic and not focused to biological problems which may also confuse the biologist.

Another drawback with many current applications is that they are desktop-based while there is a current trend in software development to switch from desktop to web-based applications for a variety of reasons including flexibility, maintenance and platform independence. Thus, although there are certain web-based tools, based on Java^TM^ webstart technology (VANTED, [42]) or related technologies (VisANT, [13]), they still have limitations similar to the ones described for certain desktop applications, and mainly the lack of a small but adequate set of bundled background databases and a relatively simplistic interface which does not require prior training. The KUPNetViz application, although simplistic in its basic concepts, aspires to initiate a set of domain-specific integrative visualization tools that focus in comprehensive interfaces. These interfaces could be used to answer biological questions from the first usages, without demanding from the user to go through long manuals, time-consuming database and literature searches to annotate the various entities in the network and understand basic mathematical concepts of graph theory.

Even though the developers of several biological network visualization and data integration packages have put substantial effort in the simplicity and clarity of the outcome, some of them can only be fully exploited by trained bioinformaticians while others require prior training in the form of workshops or seminars in order to be used at their full potential. The development of the KUPNetViz application was a continuous interaction between computer scientists and renal biologists, where several features were added, removed or re-implemented in the basis of the arguments that the application should remain simple to use, self explanatory and it should provide simple biological network snapshots. As a result, the tool manages to keep the network display and the functionalities as simple as possible by hiding and/or automating several technical aspects and mathematical properties of the biological graph. At the same time, it does not restrain more advanced users from exploring other possible mathematical properties of the resulting networks as it allows their export in text formats that can be imported to numerous existing graph analysis packages for further analysis. Additionally, taking into account a recent trend in the development of bioinformatics tools [49], which focuses to the application of user-centered design methods, the KUPNetViz comprises a good example of such a strategy, given the time that was required for the case studies to be completed and the feedback from biologists that used the application.

To our knowledge, one of the novelties of the KUPNetViz, rare among other biological network visualization tools, is the ability to visualize gene-to-gene relationships in a multispecies manner, while at the same time maintaining the simple minimalistic network display. The majority of current packages focuses either on the alignment of known biochemical networks among different species (e.g. Osprey, [50] or VANLO, [51]), allowing the user to visualize but not automatically extrapolate possible interactions that may apply across species, or on the manual building of such inter-species interaction networks based on multiple available data sources (e.g. Biological Networks, [41]). In addition, the multiple network alignment or the manual network building is usually performed with a certain cost in application and visualization simplicity, often discouraging the simple or the hurried user.

The KUPNetViz goes one step further by allowing the possibility of extrapolation of biomolecular relationships from one species to another while at the same time maintaining its straightforward visual interface and graph views (Additional file 1). This creates the unique possibility to combine –omics data from different animal models and/or human disease and quickly screen for reasonable hypotheses. As the KUPNetViz supported organisms are not equally extensively annotated in the literature (e.g. mouse is better annotated for developmental functions than human [52]), the multi-species functionality allows the extrapolation of gene-to-gene relationships and gene/protein expression data from one organism to another. While some relationships between molecules may differ between species, most relationships and biological pathways can be considered evolutionary conserved and thus allow the easy formulation of extrapolated hypotheses, regarding both biomolecular relationships and gene/protein expression under certain pathological conditions across species. The researcher can then judge the fundamental validity of these hypotheses and proceed either by rejecting a hypothesis and formulate another, or by creating a list of plausible hypothesis which can ultimately be verified in the lab.

**Figure 4:**
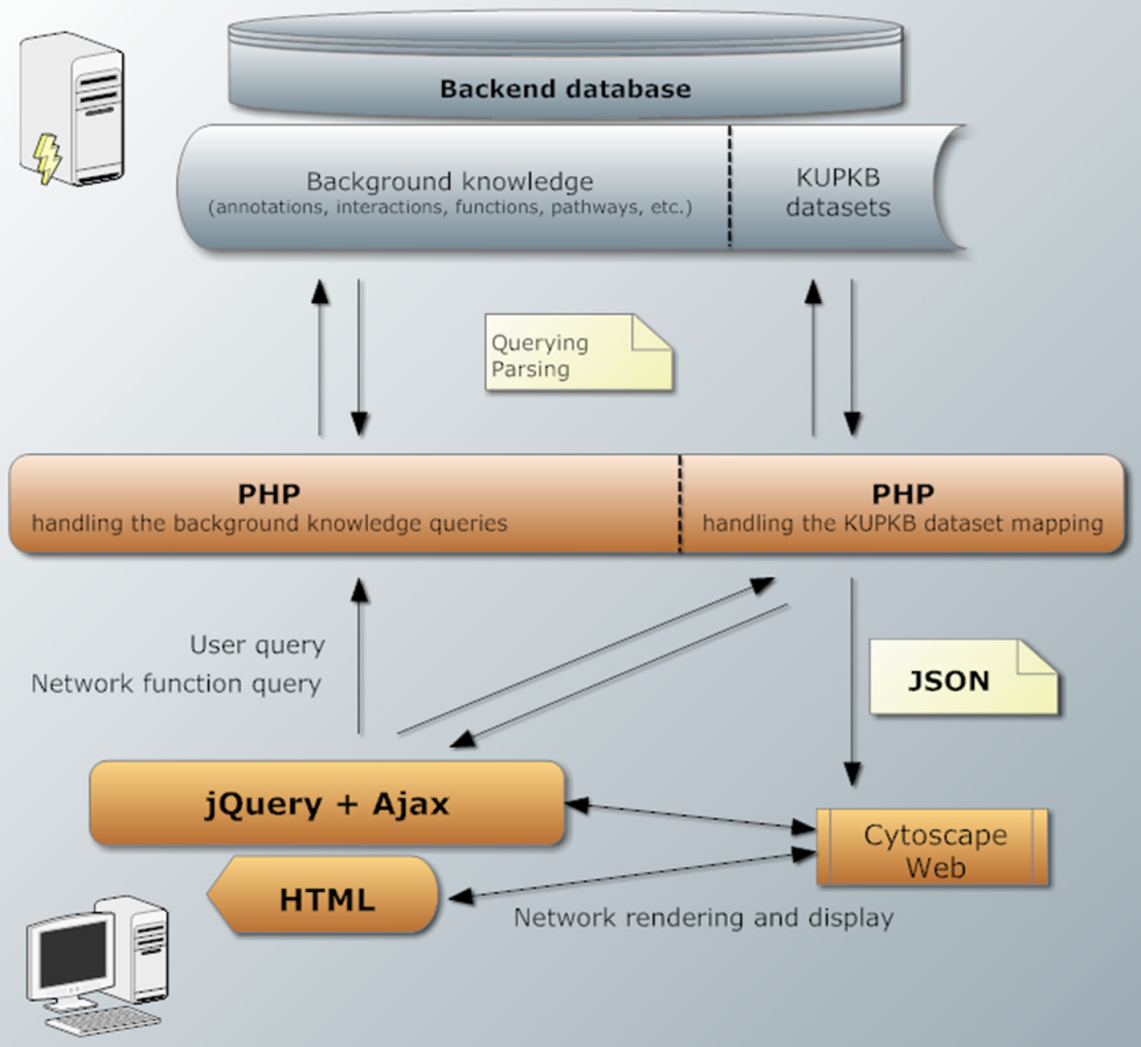
**The modularization of KUPNetViz.** A simple description of the architecture behind the KUPNetViz which consists of three simple layers, present in many similar applications: i) the background database, ii) the server PHP code layer handling database querying and parsing iii) the client side of the application where the interaction with the user is taking place. Client–server data exchange is performed using the widely used JavaScript Object Notation (JSON) format. The vertical dashed lines in the data stored in the backend database layer and the PHP layer mark the distinction of the different modules handling the background knowledge and network reconstruction and the KUPKB datasets and their mapping to the network. This distinction allow the evolution of the KUPNetViz to a more general biological network web framework which combines bundled background knowledge on biomolecular interactions with a very simple interface designed for bench researchers wishing simple biological state snapshots and not to go deeper in other network properties.

Furthermore, as the whole application can be easily built and deployed in several machines, this modularity renders it easily adaptable to other data sources (e.g. another organ). Thus, with minor modifications, the KUPNetViz may evolve to a more general framework, suitable for organ or tissue specific knowledge bases, which at the same time is not as generic as Cytoscape Web itself but includes the basic bundled knowledge to function as an independent biological network visualization application. Although such an application is not alone in its kind, it manages to keep simplicity, visual complexity and speed at acceptable levels, as compared with the two aforementioned applications ( [16,41]). A schematic representation of the KUPNetViz modularization is depicted in Figure 4.

The reasoning behind the construction of a MySQL backend database to support the application instead of using the KUPKB semantic web repository includes three main arguments: firstly, during the development we sought to maintain software modularity and re-usability even though in its current version, the visualization application is adjusted to kidney. Thus, we considered that an RDB model (especially regarding the background knowledge part) can be adjusted easier to other similar experimental data sources (e.g. a different organ) or data sources of different structure (e.g. multi-omics data from different types of cancer), requiring minor modifications only in the user interface part. Secondly, not all the background knowledge data sources that are used by the KUPNetViz were initially incorporated in the semantic web model (e.g. the protein-protein interactions or the reference pathways). This does not suggest a KUPKB model design problem but rather highlights the different initial purpose of the model which was not network visualization, but the detailed knowledge representation of kidney anatomies and disease models and the experimental data mapping and annotation to this knowledge representation model. Finally, during the design process, we realized that not all of the knowledge stored in the semantic web repository (e.g. the highly detailed localization and hierarchization of cell types and kidney tissues) was required for the minimalistic approach promoted by KUPNetViz. Thus, we used an RDB model to reduce query complexities and running speeds. In addition to the latter, the backend database building time, including the download and parsing of the background knowledge files and the re-parsing of the experimental data requires less than three hours while the rebuild of the semantic web repository requires over a day (the times were measured on the same computer). This allows the easier maintenance of the KUPNetViz and the more frequent synchronization of the backend database with the latest releases of the incorporated background knowledge sources.

## Conclusions and future work

KUPNetViz is a user-friendly biological network visualization tool dedicated to renal research. This tool uses data gathered from multiple resources on several renal pathological states and background knowledge elements regarding biomolecular interactions as well as functional and biochemical pathway associations and creates biological network snapshots that can shed light on mechanisms involved in renal disease. It provides renal biologists with an alternative network data representation, complementary to the functionality of the iKUP exploration tool with a small cost regarding the display complexity. Moreover, maybe its most important feature is the biomolecular interaction extrapolation across species which allows the researcher to quickly formulate and screen several hypotheses in a simple manner. We have demonstrated the value of KUPNetViz in two case studies, further investigating the role of calreticulin as a key player in a gene network mostly up-regulated in renal graft rejection and newly investigating the potential involvement of IL6 and the IL6 receptor in the progression of polycystic kidney disease.

The value of the KUPNetViz in kidney research will be increased in parallel with the number of related public multi-omics datasets which will be also available in the KUPKB. The expertise of the KUPNetViz hosting group in renal biology guarantees its constant curation and addition of related datasets, as these are getting published. This will potentially lead to increased confidence in the observed networks as additional biological evidence will eventually pile up from those constantly added datasets. The addition of datasets is easy and straightforward, since data are entered in ready-to-fill Excel sheets by biologists and incorporated by a single command line. We are aiming to the inclusion of the latest omics data sets in the KUPKB at least once a year from which the KUPNetViz will directly benefit from. Future extensions include the incorporation of additional biomolecule annotation and interaction resources (KEGG compounds) and the addition of a module that will identify highly enriched and over-represented biological functions and biochemical pathways using the StRAnGER algorithm [53].

## Availability and requirements

**Home page**: Accessible through the website of KUPKB and the iKUP browser (tab “KUPKB Network Visualizer”) at http://www.kupkb.org or directly at http://www.kupkb.org/vis/index.php.

**Operating system**: KUPNetViz is a web-based application thus it is platform independent.

**Requirements**: KUPNetViz is best used and viewed under Internet Explorer 8 or higher, Mozilla Firefox, Google Chrome, Apple Safari and Opera. The use of Internet Explorer 7 and lower is not recommended.

**License**: KUPNetViz is free for academic use but requires a license from the authors for any commercial purposes. The software is available without user registration.

**Further information**: An analytical user’s guide coupled with usage examples is available at the application’s homepage and as supplementary material online (Additional file 2).

## Abbreviations

KUP: Kidney and urinary pathways; KUPKB: Kidney and urinary knowledge base; KUPNetViz: Kidney and urinary pathway network visualizer; GO: Gene ontology; KEGG: Kyoto encyclopedia of genes and genomes; PKD: Polycystic kidney disease; CKD: Chronic kidney disease; IL6: Interleukin 6; IL6R: Interleukin 6 receptor; RDB: Relational database.

## Competing interest

The authors do not declare any conflicts of interest.

## Authors' contributions

PM conceived, designed and implemented the software as a whole, tested the software and drafted the manuscript. JK and JPS tested the software, suggested interface modifications, performed the case studies and contributed in drafting the manuscript. SJ and RS contributed to the database design and the integration of KUPNetViz to the iKUP browser. JB contributed in drafting the manuscript and data management. All authors have read, revised and approved the manuscript.

## Supplementary Material

Additional file 1 **An example of KUPNetVis multispecies view.** Three snapshots of a simple KUPNetViz network presenting the multispecies view, either with ‘supergenes’ encompassing orthologs among the supported species, or with collapsed ‘supergenes’ for simplified view. Click here for file

Additional file 2 **The KUPNetVis user guide.** An extensive user guide presenting all the features of the application with additional examples. Click here for file

## References

[B1] Yauk CL Rowan-Carroll A Stead JD Williams A Cross-platform analysis of global microRNA expression technologies BMC Genomics 2010 11 330 10.1186/1471-2164-11-330 20504329 PMC2890562

[B2] Kingsmore SF Multiplexed protein measurement: technologies and applications of protein and antibody arrays Nat Rev Drug Discov 2006 5 4 310 320 10.1038/nrd2006 16582876 PMC1780251

[B3] Metzker ML Sequencing technologies - the next generation Nat Rev Genet 2010 11 1 31 46 10.1038/nrg2626 19997069

[B4] Dettmer K Aronov PA Hammock BD Mass spectrometry-based metabolomics Mass Spectrom Rev 2007 26 1 51 78 10.1002/mas.20108 16921475 PMC1904337

[B5] Harding SD Armit C Armstrong J Brennan J Cheng Y Haggarty B Houghton D Lloyd-MacGilp S Pi X Roochun Y The GUDMAP database–an online resource for genitourinary research Development 2011 138 13 2845 2853 10.1242/dev.063594 21652655 PMC3188593

[B6] Miyamoto M Yoshida Y Taguchi I Nagasaka Y Tasaki M Zhang Y Xu B Nameta M Sezaki H Cuellar LM In-depth proteomic profiling of the normal human kidney glomerulus using two-dimensional protein prefractionation in combination with liquid chromatography-tandem mass spectrometry J Proteome Res 2007 6 9 3680 3690 10.1021/pr070203n 17711322

[B7] Pisitkun T Bieniek J Tchapyjnikov D Wang G Wu WW Shen RF Knepper MA High-throughput identification of IMCD proteins using LC-MS/MS Physiol Genomics 2006 25 2 263 276 10.1152/physiolgenomics.00214.2005 16449382 PMC1436036

[B8] Klein J Jupp S Moulos P Fernandez M Buffin-Meyer B Casemayou A Chaaya R Charonis A Bascands JL Stevens R The KUPKB: a novel Web application to access multiomics data on kidney disease FASEB J 2012 26 5 2145 2153 10.1096/fj.11-194381 22345404

[B9] Antezana E Kuiper M Mironov V Biological knowledge management: the emerging role of the Semantic Web technologies Brief Bioinform 2009 10 4 392 407 10.1093/bib/bbp024 19457869

[B10] Bonneau R Learning biological networks: from modules to dynamics Nat Chem Biol 2008 4 11 658 664 10.1038/nchembio.122 18936750

[B11] Zhu X Gerstein M Snyder M Getting connected: analysis and principles of biological networks Genes Dev 2007 21 9 1010 1024 10.1101/gad.1528707 17473168

[B12] Suderman M Hallett M Tools for visually exploring biological networks Bioinformatics 2007 23 20 2651 2659 10.1093/bioinformatics/btm401 17720984

[B13] Hu Z Hung JH Wang Y Chang YC Huang CL Huyck M DeLisi C VisANT 3.5: multi-scale network visualization, analysis and inference based on the gene ontology Nucleic Acids Res 2009 37 Web Server issue 115 121 10.1093/nar/gkp406 PMC2703932 19465394

[B14] Gehlenborg N O'Donoghue SI Baliga NS Goesmann A Hibbs MA Kitano H Kohlbacher O Neuweger H Schneider R Tenenbaum D Visualization of omics data for systems biology Nat Methods 2010 7 3 Suppl S56 S68 20195258 10.1038/nmeth.1436

[B15] Pavlopoulos GA Wegener AL Schneider R A survey of visualization tools for biological network analysis BioData Min 2008 1 12 10.1186/1756-0381-1-12 19040716 PMC2636684

[B16] Paquette J Tokuyasu T EGAN: exploratory gene association networks Bioinformatics 2010 26 2 285 286 10.1093/bioinformatics/btp656 19933825 PMC2804305

[B17] Lopes CT Franz M Kazi F Donaldson SL Morris Q Bader GD Cytoscape Web: an interactive web-based network browser Bioinformatics 2010 26 18 2347 2348 10.1093/bioinformatics/btq430 20656902 PMC2935447

[B18] Jupp S Klein J Schanstra J Stevens R Developing a kidney and urinary pathway knowledge base J Biomed Semantics 2011 2 Suppl 2 S7 10.1186/2041-1480-2-S2-S7 21624162 PMC3102896

[B19] Consortium TU Reorganizing the protein space at the Universal Protein Resource (UniProt) Nucleic Acids Res 2012 40 Database issue 71 75 10.1093/nar/gkr981 PMC3245120 22102590

[B20] Flicek P Amode MR Barrell D Beal K Brent S Carvalho-Silva D Clapham P Coates G Fairley S Fitzgerald S Ensembl 2012 Nucleic Acids Res 2012 40 Database issue 84 90 10.1093/nar/gkr991 PMC3245178 22086963

[B21] Kozomara A Griffiths-Jones S miRBase: integrating microRNA annotation and deep-sequencing data Nucleic Acids Res 2011 39 Database issue 152 157 10.1093/nar/gkq1027 PMC3013655 21037258

[B22] Guberman JM Ai J Arnaiz O Baran J Blake A Baldock R Chelala C Croft D Cros A Cutts RJ BioMart Central Portal: an open database network for the biological community Database (Oxford) 2011 2011 bar041 10.1093/database/bar041 21930507 PMC3263598

[B23] Szklarczyk D Franceschini A Kuhn M Simonovic M Roth A Minguez P Doerks T Stark M Muller J Bork P The STRING database in 2011: functional interaction networks of proteins, globally integrated and scored Nucleic Acids Res 2011 39 Database issue 561 568 10.1093/nar/gkq973 PMC3013807 21045058

[B24] Kanehisa M Goto S Sato Y Furumichi M Tanabe M KEGG for integration and interpretation of large-scale molecular data sets Nucleic Acids Res 2012 40 Database issue 109 114 10.1093/nar/gkr988 PMC3245020 22080510

[B25] Secrier M Pavlopoulos GA Aerts J Schneider R Arena3D: visualizing time-driven phenotypic differences in biological systems BMC Bioinformatics 2012 13 45 10.1186/1471-2105-13-45 22439608 PMC3368716

[B26] Kypreou KP Kavvadas P Karamessinis P Peroulis M Alberti A Sideras P Psarras S Capetanaki Y Politis PK Charonis AS Altered expression of calreticulin during the development of fibrosis Proteomics 2008 8 12 2407 2419 10.1002/pmic.200700831 18563736

[B27] Abrass CK Berfield AK Stehman-Breen C Alpers CE Davis CL Unique changes in interstitial extracellular matrix composition are associated with rejection and cyclosporine toxicity in human renal allograft biopsies Am J Kidney Dis 1999 33 1 11 20 10.1016/S0272-6386(99)70252-0 9915262

[B28] Mas V Maluf D Archer K Yanek K Mas L King A Gibney E Massey D Cotterell A Fisher R Establishing the molecular pathways involved in chronic allograft nephropathy for testing new noninvasive diagnostic markers Transplantation 2007 83 4 448 457 10.1097/01.tp.0000251373.17997.9a 17318078

[B29] Siddiqui I Khan ZA Lian D Jiang J Zhong R Garcia B Chakrabarti S Endothelin-mediated oncofetal fibronectin expression in chronic allograft nephropathy Transplantation 2006 82 3 406 414 10.1097/01.tp.0000228905.44649.06 16906041

[B30] Natsume M Tsuji H Harada A Akiyama M Yano T Ishikura H Nakanishi I Matsushima K Kaneko S Mukaida N Attenuated liver fibrosis and depressed serum albumin levels in carbon tetrachloride-treated IL-6-deficient mice J Leukoc Biol 1999 66 4 601 608 10534116

[B31] Yoshizaki A Yanaba K Ogawa A Asano Y Kadono T Sato S Immunization with DNA topoisomerase I and Freund's complete adjuvant induces skin and lung fibrosis and autoimmunity via interleukin-6 signaling Arthritis Rheum 2011 63 11 3575 3585 10.1002/art.30539 21792823

[B32] Zhang W Wang W Yu H Zhang Y Dai Y Ning C Tao L Sun H Kellems RE Blackburn MR Interleukin 6 underlies angiotensin II-induced hypertension and chronic renal damage Hypertension 2012 59 1 136 144 10.1161/HYPERTENSIONAHA.111.173328 22068875 PMC3842011

[B33] O'Reilly S Ciechomska M Cant R Hugle T van Laar JM Interleukin-6, its role in fibrosing conditions Cytokine Growth Factor Rev 2012 23 3 99 107 10.1016/j.cytogfr.2012.04.003 22561547

[B34] Uawithya P Pisitkun T Ruttenberg BE Knepper MA Transcriptional profiling of native inner medullary collecting duct cells from rat kidney Physiol Genomics 2008 32 2 229 253 17956998 10.1152/physiolgenomics.00201.2007 PMC2276652

[B35] Song X Di Giovanni V He N Wang K Ingram A Rosenblum ND Pei Y Systems biology of autosomal dominant polycystic kidney disease (ADPKD): computational identification of gene expression pathways and integrated regulatory networks Hum Mol Genet 2009 18 13 2328 2343 10.1093/hmg/ddp165 19346236

[B36] Koupepidou P Felekkis KN Kranzlin B Sticht C Gretz N Deltas C Cyst formation in the PKD2 (1–703) transgenic rat precedes deregulation of proliferation-related pathways BMC Nephrol 2010 11 23 10.1186/1471-2369-11-23 20813037 PMC2936873

[B37] Menon V Rudym D Chandra P Miskulin D Perrone R Sarnak M Inflammation, oxidative stress, and insulin resistance in polycystic kidney disease Clin J Am Soc Nephrol 2011 6 1 7 13 10.2215/CJN.04140510 20829421 PMC3022250

[B38] Lai X Bacallao RL Blazer-Yost BL Hong D Mason SB Witzmann FA Characterization of the renal cyst fluid proteome in autosomal dominant polycystic kidney disease (ADPKD) patients Proteomics Clin Appl 2008 2 7–8 1140 1152 20411046 10.1002/prca.200780140 PMC2857342

[B39] Mason SB Lai X Bacallao RL Blazer-Yost BL Gattone VH Wang KC Witzmann FA The biomarker enriched proteome of autosomal dominant polycystic kidney disease cyst fluid Proteomics Clin Appl 2009 3 10 1247 1250 10.1002/prca.200800163 20526430 PMC2880522

[B40] Chen WC Tzeng YS Li H Gene expression in early and progression phases of autosomal dominant polycystic kidney disease BMC Res Notes 2008 1 131 10.1186/1756-0500-1-131 19099603 PMC2632667

[B41] Kozhenkov S Dubinina Y Sedova M Gupta A Ponomarenko J Baitaluk M BiologicalNetworks 2.0--an integrative view of genome biology data BMC Bioinformatics 2010 11 610 10.1186/1471-2105-11-610 21190573 PMC3019228

[B42] Junker BH Klukas C Schreiber F VANTED: a system for advanced data analysis and visualization in the context of biological networks BMC Bioinformatics 2006 7 109 10.1186/1471-2105-7-109 16519817 PMC1413562

[B43] Dinkla K Westenberg MA van Wijk JJ Compressed adjacency matrices: untangling gene regulatory networks IEEE Trans Visualization and Computer Graphics 2012 18 12 2457 2466 10.1109/TVCG.2012.208 26357154

[B44] Freeman TC Goldovsky L Brosch M van Dongen S Maziere P Grocock RJ Freilich S Thornton J Enright AJ Construction, visualisation, and clustering of transcription networks from microarray expression data PLoS Comput Biol 2007 3 10 2032 2042 17967053 10.1371/journal.pcbi.0030206 PMC2041979

[B45] Forman JJ Clemons PA Schreiber SL Haggarty SJ SpectralNET–an application for spectral graph analysis and visualization BMC Bioinformatics 2005 6 260 10.1186/1471-2105-6-260 16236170 PMC1276787

[B46] Auber D Mutzel P, Junger M A huge graph visualization framework Graph Drawing Software 2004 Heidelberg, Germany: Springer 105 126

[B47] Prinz S Avila-Campillo I Aldridge C Srinivasan A Dimitrov K Siegel AF Galitski T Control of yeast filamentous-form growth by modules in an integrated molecular network Genome Res 2004 14 3 380 390 10.1101/gr.2020604 14993204 PMC353223

[B48] Batagelj V Mrvar A Pajek - Program for Large Network Analysis Connections 1998 21 2 47 57

[B49] de Matos P Cham JA Cao H Alcantara R Rowland F Lopez R Steinbeck C The Enzyme Portal: a case study in applying user-centred design methods in bioinformatics BMC Bioinformatics 2013 14 103 10.1186/1471-2105-14-103 23514033 PMC3623738

[B50] Breitkreutz BJ Stark C Tyers M Osprey: a network visualization system Genome Biol 2003 4 3 R22 10.1186/gb-2003-4-3-r22 12620107 PMC153462

[B51] Brasch S Linsen L Fuellen G VANLO–interactive visual exploration of aligned biological networks BMC Bioinformatics 2009 10 327 10.1186/1471-2105-10-327 19821976 PMC2766391

[B52] da Huang W Sherman BT Lempicki RA Bioinformatics enrichment tools: paths toward the comprehensive functional analysis of large gene lists Nucleic Acids Res 2009 37 1 1 13 10.1093/nar/gkn923 19033363 PMC2615629

[B53] Chatziioannou AA Moulos P Exploiting statistical methodologies and controlled vocabularies for prioritized functional analysis of genomic experiments: the StRAnGER Web application Front Neurosci 2011 5 8 21293737 10.3389/fnins.2011.00008 PMC3032379

