# Supplementary material for: The KUPNetViz: a biological network viewer for multiple -omics datasets in kidney diseases
Source: BMC Bioinformatics. 2013 Jul 24;14:235. doi: 10.1186/1471-2105-14-235 (PMC3725151; doi:10.1186/1471-2105-14-235)
Supplement: Additional file 1 — An example of KUPNetVis multispecies view. Three snapshots of a simple KUPNetViz network presenting the multispecies view, either with ‘supergenes’ encompassing orthologs among the supported species, or with collapsed ‘supergenes’ for simplified view. [file 1471-2105-14-235-S1.pdf]

A

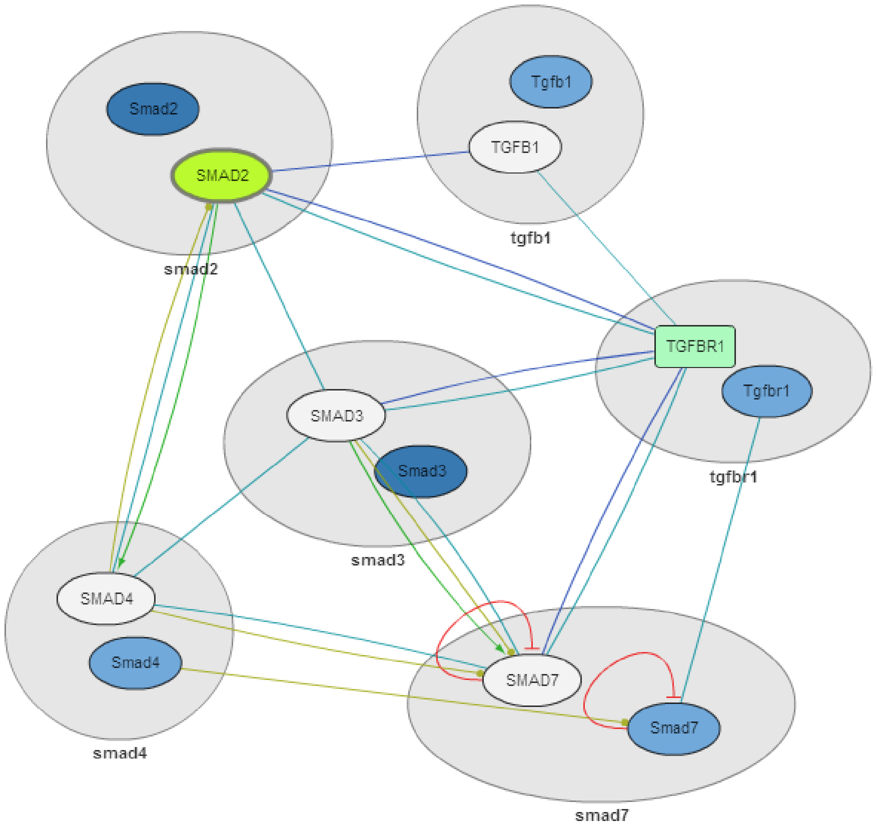

Smad2
SMAD2
smad2
Tgfb1
TGFB1
tgfb1
TGFBR1
Tgfbr1
tgfbr1
SMAD3
Smad3
smad3
SMAD4
Smad4
smad4
SMAD7
Smad7
smad7

B

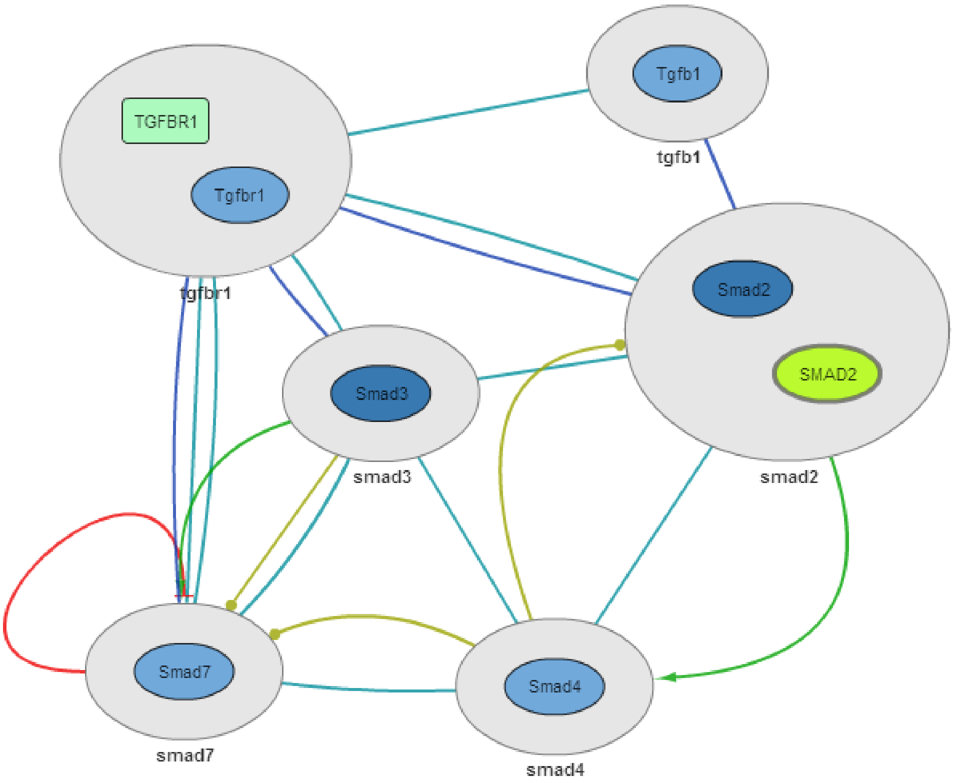

TGFBR1
Tgfbr1
tgfbr1
Tgfb1
tgfb1
Smad2
SMAD2
smad2
Smad3
smad3
Smad7
smad7
Smad4
smad4

C

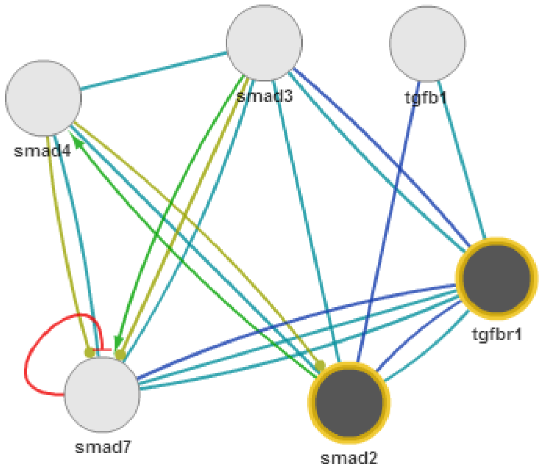

Supplemental figure 1: Example of multi-species view in the KUPNetViz.

Orthologous genes are represented as compound nodes (‘supergenes’) in two possible ways: either as compound nodes encompassing single organism orthologs (A) or as simple compound nodes with hidden orthologs to reduce the complexity of the snapshot (B, C). In the first case (A), gene-to-gene relationships are organism specific while in the second case they are depicted as supergene-to-supergene relationships (‘superedges’, B, C). In the case of colored or multiple-colored nodes based on KUPKB expression data mapping to the network, the complexity can be further reduced by collapsing the colored nodes to a single colored node (C), when working in multi-species mode.
