# Supplementary material for: The KUPNetViz: a biological network viewer for multiple -omics datasets in kidney diseases
Source: BMC Bioinformatics. 2013 Jul 24;14:235. doi: 10.1186/1471-2105-14-235 (PMC3725151; doi:10.1186/1471-2105-14-235)
Supplement: Additional file 2 — The KUPNetVis user guide. An extensive user guide presenting all the features of the application with additional examples. [file 1471-2105-14-235-S2.pdf]

KUPNetViz

Kidney and Urinary Pathway Network Visualizer

version 1.0

User's manual

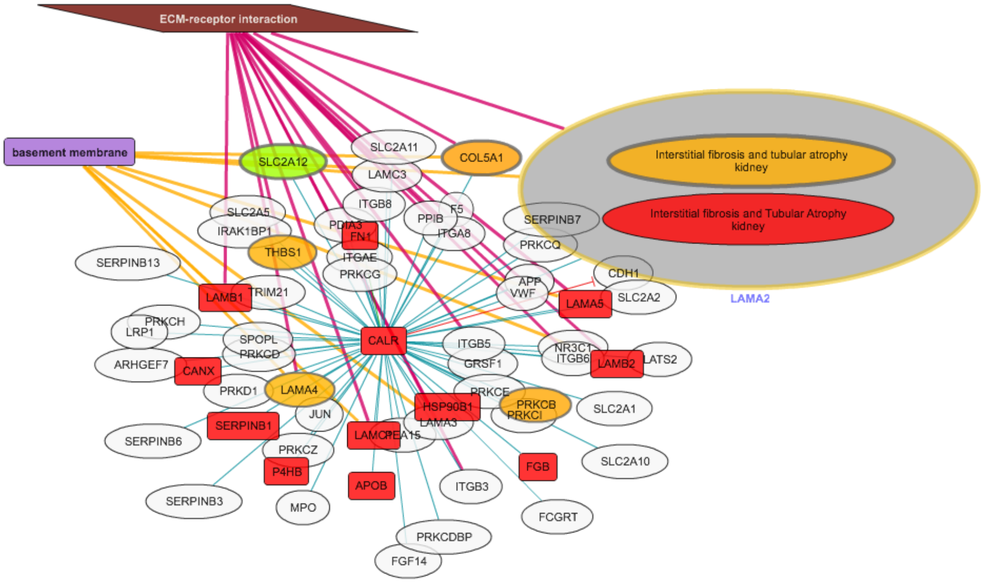

Institute of Cardiovascular and Metabolic Disease – I2MC

Institut National de la Santé et de la Recherche Médicale (INSERM)

©2012

Contents

Contents 2
1. Overview 4
1.1. Description 4
1.2. Launching the KUPNetViz 4
1.3. How to use this guide 4
1.4. Issue reporting 5
1.5. Quick start 5
1.5.1. Functionalities at a glance 5
1.5.2. A complete example 7
2. Using the KUPNetViz in single species mode 10
2.1. Searching for genes/proteins/miRNAs 11
2.2. Network description 11
2.2.1. Network initialization 11
2.2.2. Node and edge description 12
2.3. Network functionalities 14
2.3.1. 1st and 2nd level neighbors 14
2.3.2. Hiding nodes/edges 16
2.3.3. Deleting nodes/edges 17
2.3.4. Restoring the network 17
2.4. Element annotation 17
2.4.1. Node annotation 18
2.4.2. Edge annotation 19
2.5. Adding KUPKB data 20
2.5.1. The KUPKB gene/protein data panel 20
2.5.2. The KUPKB miRNA data panel 24
2.5.3. The Displayed interactions panel 25
2.5.4. Displaying neighbors 26
2.6. Adding functional and pathway data 26
2.6.1. The Gene Ontology panel 26
2.6.2. The KEGG pathways panel 27
2.6.3. The miRBase microRNA panel 29
2.7. Advanced options 30
2.7.1. The Search options panel 30

2.7.2. The Coloring options panel 32
2.7.3. Other advanced options 34
3. Using the KUPNetViz in multiple species mode 36
3.1. Searching for genes/proteins/miRNAs 36
3.2. Network description 36
3.2.1. Network initialization 36
3.2.2. Node and edge description 36
3.3. Network functionalities 37
3.4. Element annotation 37
3.5. Adding KUPKB data 37
3.6. Adding functional and pathway data 38
3.7. Advanced options 38
4. View and Export 39
4.1. View 39
4.2. Export 39
Appendix A 41

1. Overview

1.1. Description

Recently, we presented the Kidney and Urinary Pathway Knowledge Base (KUPKB), a publicly available repository which organizes an important amount of existing knowledge regarding renal tissue, cell and disease categorization, using Semantic Web technologies in order to describe and unify data in a reusable integrative manner. The KUPKB currently contains over 220 manually curated, annotated and in some cases reanalyzed multi-omics datasets derived from renal research studies, which can be queried through a user-friendly search interface named iKUP and accessible at http://www.kupkb.org.

The KUPNetViz is an interactive biological network visualization application, which at the same time comprises a querying and exploration tool complementary to the iKUP. Its aims at providing to KUP scientists a way to extend their research by combining the results of iKUP with a more generalized image depicting interactions among the queried molecules and their genomic neighbors, coupled with functional and biochemical pathway annotation. The KUPNetViz uses the Cytoscape Web library for graph rendering (http://cytoscapeweb.cytoscape.org/).

1.2. Launching the KUPNetViz

The KUPNetViz is a web application and thus, it does not need a local installation. You can use only your web browser, navigate to http://www.kupkb.org and from there select the link to the KUPNetViz. Alternatively, you can navigate directly to http://www.kupkb.org/viz/index.php. Another way to launch the KUPNetViz is to firstly query the molecules of your preference to the iKUP and then press the relative button in the iKUP application (soon to become available). This will cause KUPNetViz to launch with a network initialized with the molecules you queried in iKUP. For best navigating and viewing experience, please use KUPNetViz with Mozilla Firefox, Google Chrome, or Internet Explorer 8 or higher. KUPNetViz has not been extensively tested with Internet Explorer versions lower than 8, but it is most likely that the interface will not be displayed correctly.

1.3. How to use this guide

This guide is written on a specific example basis, much like a tutorial. This means that in the beginning of section 2, you will initialize the application using a given list of genes, proteins and miRNAs and the rest of the guide after that point will explain the application’s functionalities based on this example and referring to nodes and edges that are derived from this example where possible. In this way, you can learn how to use the KUPNetViz in action.

1.4. Issue reporting

The KUPNetViz is a new tool developed and tested by a very small group of people. This means that it has not been extensively tested and minor problems might still exist. If you notice something unusual such error messages, wrong colors or wrong relationships among nodes do not hesitate to report this behavior to pmoulos@eie.gr with a small example on how to reproduce the problem and if possible, also a screenshot.

1.5. Quick start

1.5.1. Functionalities at a glance

The KUPNetViz application consists of a single web page divided in three main parts: i) the interaction network canvas, which is the Adobe Flash™ component of Cytoscape Web and displays the interaction networks, ii) the information and annotation section which contains several legends regarding the network components and a dynamic subsection which displays information and external links for the selected network elements, and iii) the application controls which are organized in four tabs containing data search and mapping options as well as more advanced application controls. The controls of the application are described in more detail below:

1. The first tab contains the search area and species selection list together with brief instructions on how the application is used.
2. The second tab contains the multi-omics expression KUPKB datasets that are associated with the queried elements and a few simple options that control the kidney disease and location criteria selection as well as the displayed interactions and the queries for neighbors of the selected nodes.
3. The third tab contains lists with meta-data (GO terms, KEGG pathways, miRNAs targeting genes/proteins in the network) associated with the queried molecules and their respective display controls.
4. Finally, the fourth tab contains more advanced options which control additional functionalities such as the search mode of genes/proteins (whole genomes or genes contained only in the expression datasets) and their interactions (all possible or only among selected molecules, interaction score threshold etc.), and colouring and annotation modes and restrictions, single or multiple kidney disease/location and dataset selection. The fourth tab also controls on how multiple species networks are displayed, either as compound nodes with separated interactions per organism or as single multispecies nodes with merged and possibly extrapolated interactions for all the selected organisms.

This configuration allows the user to easily navigate through the application and explore and map KUPKB data without leaving the main network view. The usage of the application is very simple, self-guiding and can be summarized in three basic steps:

1. Search using a molecule list of interest. The resulting network consists of simple or compound nodes ('supergenes') according to single or multiple species selection respectively and edges for the following protein-protein interactions: binding, modification, expression (stimulation), expression (inhibition) and activation. In the case of multiple species selection the edges reflect the extrapolation of the possible interactions from one species to another ('superedges'). The initial network consists only of genes and does not contain any dataset mapping. This means that the initial network consists only of simple nodes representing genes and edges representing recorded protein-protein interactions but no colouring based on expression data or any further annotation elements.
2. Mapping of experimental data to the network. The user may navigate and select through multiple renal locations and diseases associated with the queried molecules and colour the network according to one or more datasets meeting specified criteria. These criteria include combinations of kidney locations and disease models associated with these locations. Then, the available expression data are retrieved and the network can be coloured based on multiple expression data. For example, based on the recorded KUPKB datasets, the queried gene/protein "calreticulin" is associated with the kidney anatomies "bladder urine", "glomerulus", "kidney" and "kidney proximal tubule epithelial cell" and with the disease models "interstitial fibrosis and tubular atrophy" and "TGFbeta in vitro model". Then, the criteria combination disease: "TGFbeta in vitro model" and location: "kidney proximal tubule epithelial cell" is found in two expression datasets (namely "Chen, Am J Nephrol, 2010" and "Hills, Mol Endocrinol, 2010") which can be used to colour the network nodes. Molecules functionally connected to the queried ones ('neighbors' obtained by network expansion using known biological connections) can be fetched and re-queried against experimental data in the KUPKB. The location, disease and dataset list contents are dynamic, meaning that they are refilled each time a new network is created or new nodes are added (e.g. in the forms of a gene's neighbors) and restricted according to multiple selections so that specific criteria sets can be created. After the data mapping to the network, associated network nodes are assigned with additional data, specific to the mapped data, e.g. a description of the related dataset and/or publication. Multiple datasets can be mapped to a single node in the network.
3. Association with biological functions, processes and cellular components, with biochemical pathways and with miRNAs targeting the entities of interest. Association nodes are added to the network. The GO, KEGG and miRNA lists are dynamically repopulated when new gene/protein nodes are added to the network. When the researcher uses KUPNetViz in multispecies mode, the biochemical pathways are not organism specific but the KEGG reference pathways are used instead.

Clicking on a node/edge element of the network displays additional information and links to external resources (e.g. Entrez, Ensembl, GeneCards, simple PubMed searches etc.). Descriptions of the respective

experimental source (experimental description, biological conditions under investigation, species and related publication) are also available either on the fly by dynamic tooltips or in separate web pages. At any time, the user has access to advanced functionalities regarding edge filtering according to interaction type, node colouring and annotation modes, biomolecule search modes and network control (e.g. layout algorithms). Simple tooltips provide basic comprehension of the application.

1.5.2. A complete example

This section contains a quick start example in case you feel enthousiastic or just lazy. It initializes a simple, single-species network (one gene), searches its close neighbors, maps some KUPKB data on the expanded network, and associates example GO terms and KEGG pathways. This quick example explores the potential role of *calreticulin*, a well known protein involved in renal disease in animals, to human renal disease, the regulation of neighboring genes and the biological functions and pathways involved.

1. Go to http://www.kupkb.org and click on the related link that leads to KUPNetViz or visit directly http://www.kupkb.org/vis/index.php. In the search box below the main network view, type "calr" (the application supports an autosuggest and autocomplete function). Click "GO".

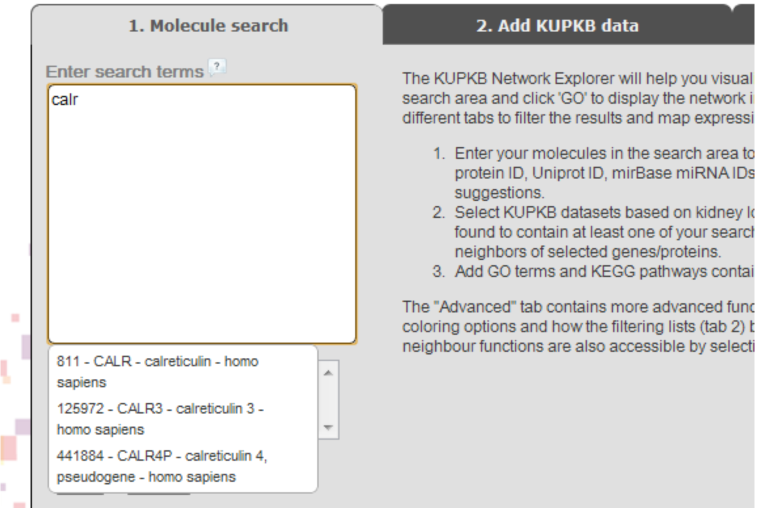

2. A node labeled "CALR" will appear in the network view. Click on the node (it will change color to cyan) and right-click on it. A submenu will appear. You should then click on "Get level 1 neighbors". The application will search for the close functional neighbors of calreticulin, display them in a network and also fetch all related KUPKB data as well as background knowledge including GO terms, KEGG pathways and miRNAs that target genes in the network. You will notice that because of the neighbors, the network has become a little complex. On the top menu of the application, click on "View"→"Help! Many genes!" item. Your network should now look like the following image:

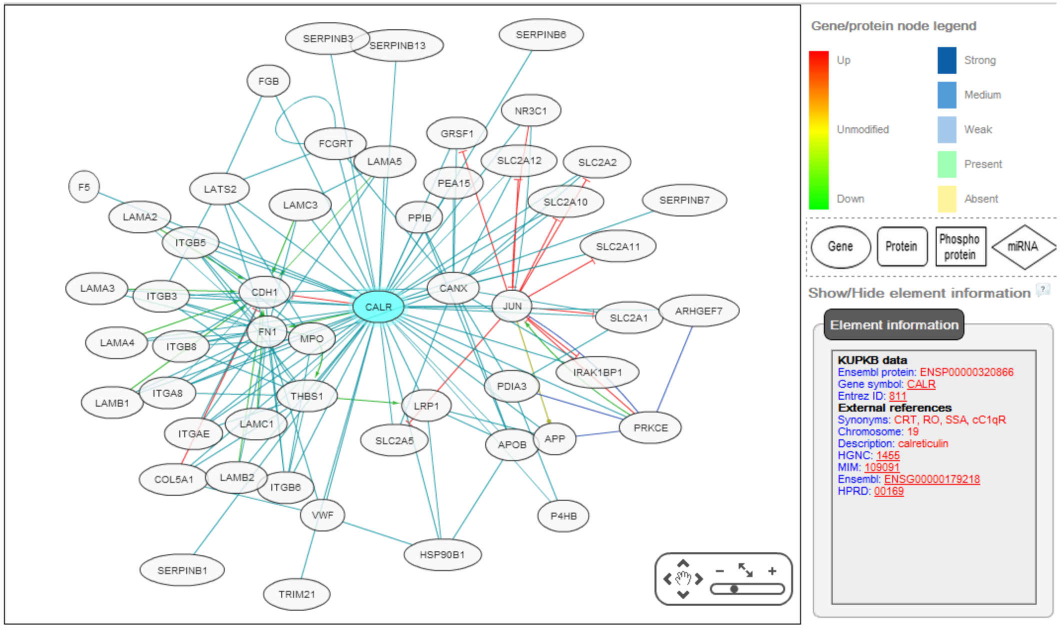

3. You will now map some KUPKB data on the created calreticulin network. Go to the 2nd tab of the application ("Add KUPKB data"). In the panel "KUPKB gene/protein data", select "Interstitial fibrosis and tubular atrophy" from the "Disease" list. You will notice that the "Location" and "Dataset lists" are filtered and that you now have 3 datasets in the "Dataset" list. Holding down the "Ctrl" key, click on all 3 of them (Nakorchevsky, J Am Soc Nephrol, 2010; Rodder, Am J Transplant, 2009; Scherer, Nephrol Dial Translpant, 2009) to select them and click on "Color network!". Your network should now look like the image below. For an explanation regarding the big grey node with the subnodes inside, look at section 2.2.2. Remember, if your network becomes too complex for any reason, there is always the "Help! Many genes!" item under the "View" menu on top of the application.

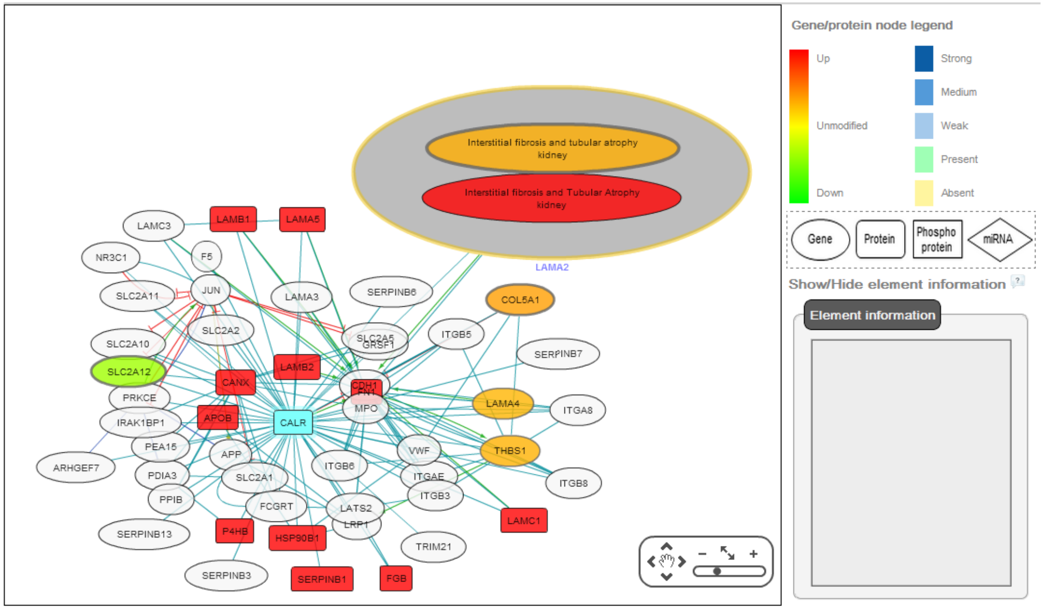

4. You will now add Gene Ontology and KEGG pathway data. Go to the 3rd tab of the application, “Add GO, KEGG or miRNA data”. In the “Gene Ontology” panel, select “basement membrane” from the Component list and click on the “Show selected” button below. Next, in the “KEGG Pathways” panel, select “ECM-receptor interaction” and click on the “Show selected” button below. Your application window should now look like the following image:

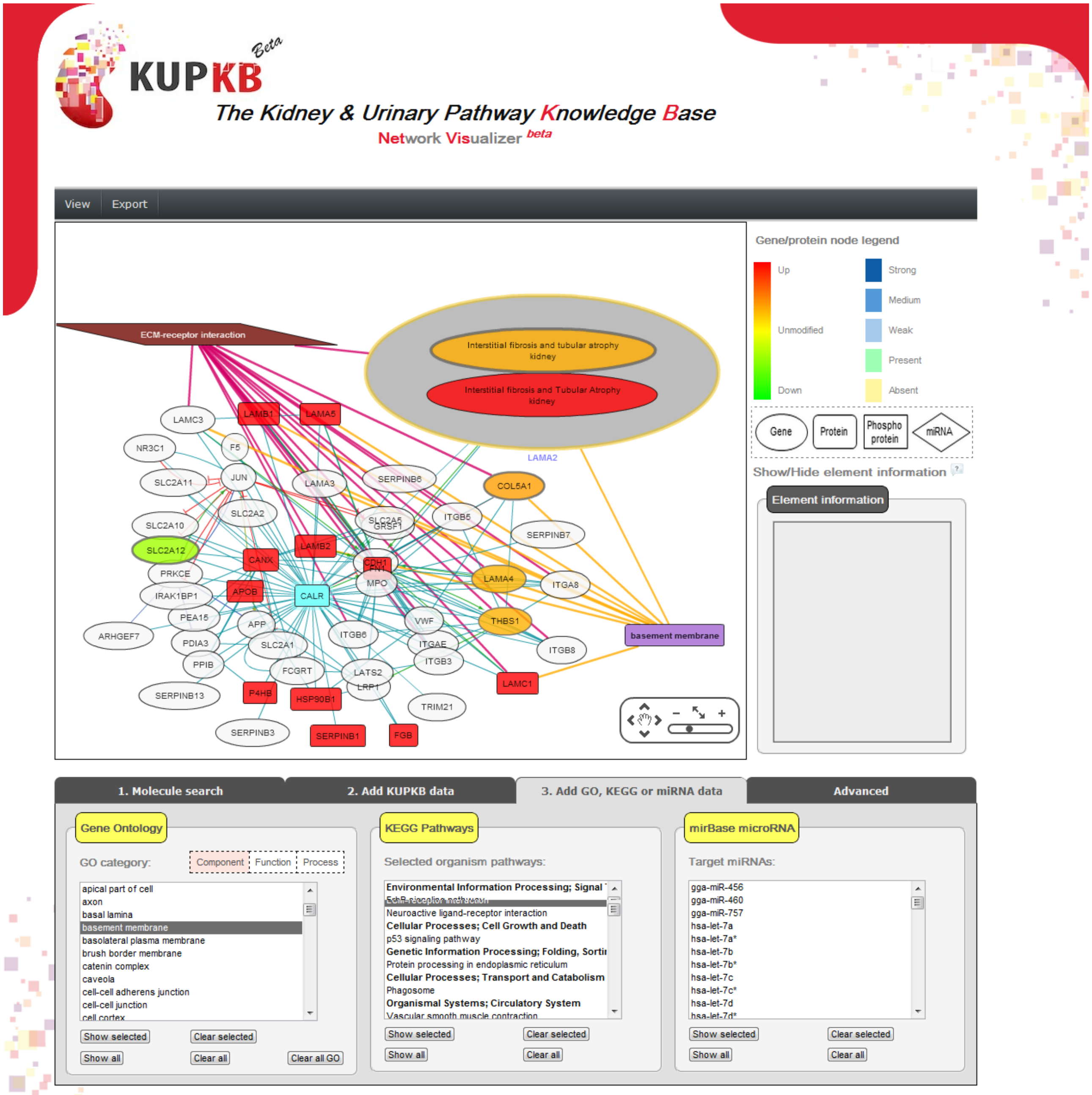

2. Using the KUPNetViz in single species mode

The KUPNetViz can be used to query for molecules either in single species mode or in multiple species mode. The latter is very easy and can be performed just by selecting more than one species in the list of species below the search field. The multiple species mode functionality will be covered in detail in section 3. The figure below displays the first page of the application with a sample network.

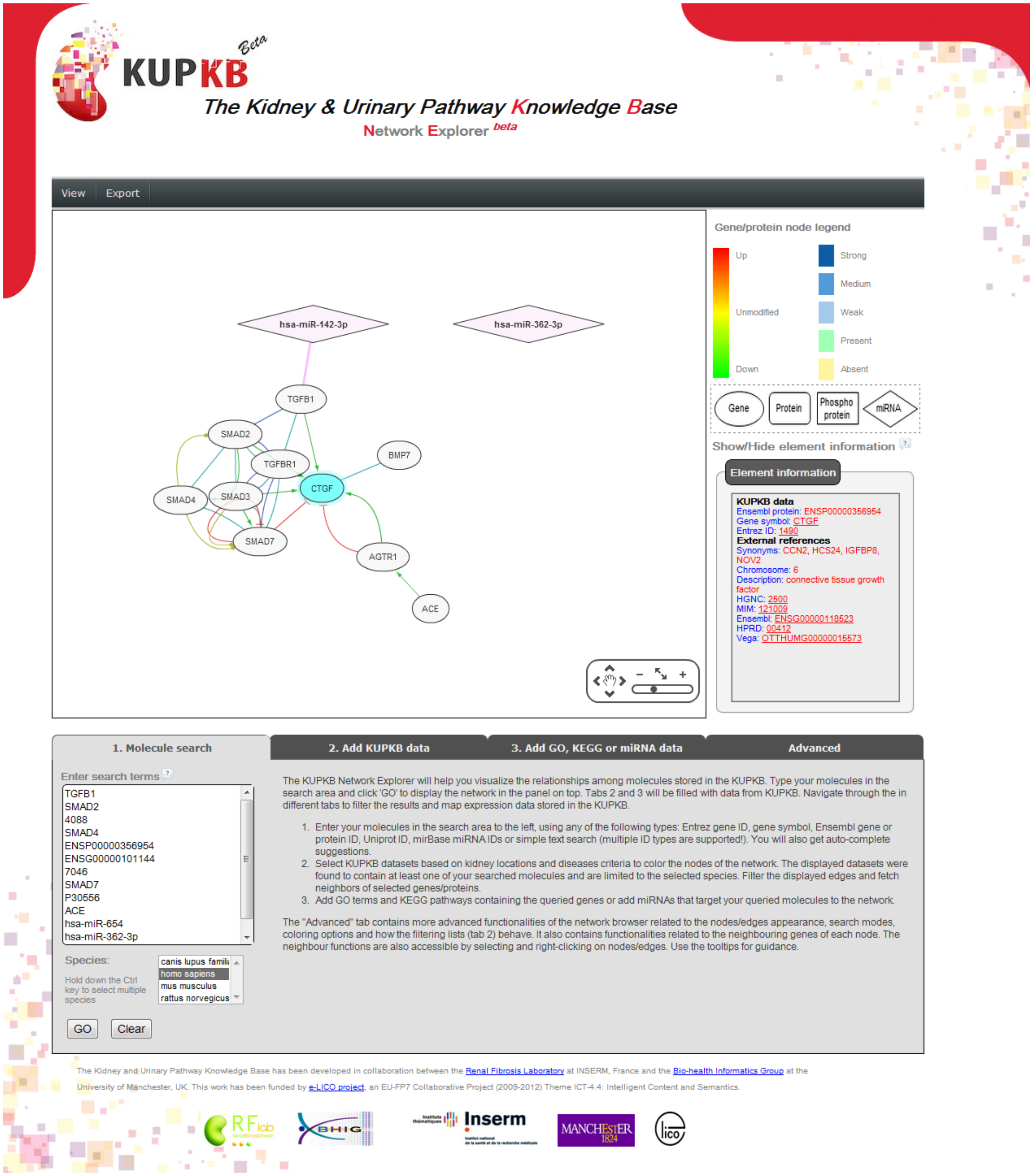

2.1. Searching for genes/proteins/miRNAs

You can search for your preferred molecules using the KUPNetViz very easily, either by typing directly in the search area (the box under "Enter search terms") or by pasting a list of identifiers. While typing, the KUPNetViz will propose you several molecules starting or containing the letters you type through its auto-complete function. You should keep in mind that you can't paste or type more than 500 molecules.

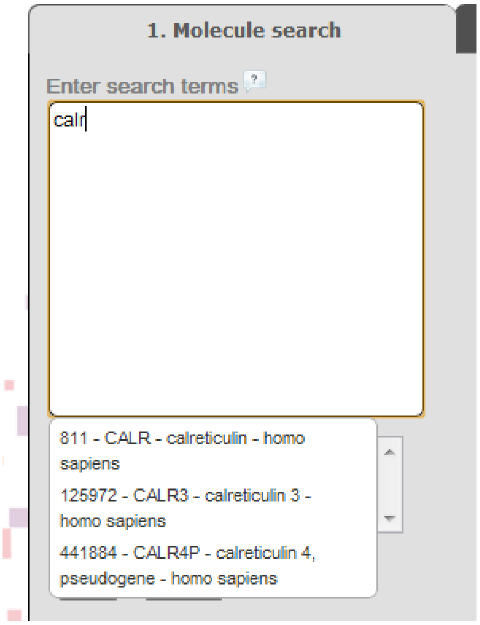

The supported gene, protein and miRNA identifiers by KUPNetViz are HUGO gene symbols (e.g. "calr"), Entrez accession numbers (e.g. 811), UniProt accessions (e.g. P30566), Ensembl gene and protein IDs (e.g. ENSG00000101144 or ENSP00000356954) and miRBase accession (e.g. hsa-miR-362-3p). You can also search by simple free text (e.g. "angiotensin II"). You can use mixed IDs as KUPNetViz automatically recognizes them.

2.2. Network description

2.2.1. Network initialization

To initialize a molecular interaction network go to the 1st tab of the application ("Molecule search"), type your preferred molecules in the search box under "Enter search terms" and click the **GO** button. Keep in mind that the initial network is based only on the background knowledge of the KUPKB (that is only genes and relationships among them). This means that when the network is initialized, you will see only the node types corresponding to genes, labeled by their HUGO gene symbol and their default color. You should also see the relationships among them as they are derived from the background knowledge stored in the KUPKB. See Appendix A for a short description of the background knowledge used to create the molecular interaction networks.

For the purposes of this guide, you should initialize a network using the identifiers in the left column of the table below. The right column contains the corresponding HUGO gene symbol for the entries in the left column.

Search term	HUGO gene symbol
TGFB1	TGFB1
SMAD2	SMAD2
4088	SMAD3
SMAD4	SMAD4
ENSP00000356954	BMP7
ENSG00000101144	CTGF
7046	TGFBR1
SMAD7	SMAD7
P30556	AGTR1
ACE	ACE
hsa-miR-362-3p	-
hsa-miR-142-3p	-

After entering the above search terms (most easily done by copying and pasting the above list) and clicking the GO button, the network should look very similar to the one presented in the application image in the beginning of section 2.

2.2.2. Node and edge description

Node shapes and colors have different meaning in KUPNetViz. Nodes representing genes/proteins/ miRNAs are colored based on abundance values present in the KUPKB. Although a quick reference regarding gene/protein/miRNA node shapes and colors is displayed on the right part of the KUPNetViz application as shown below

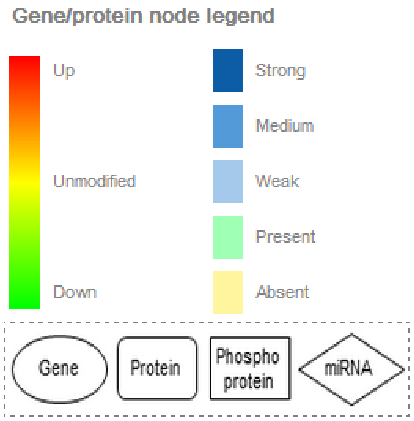

The following table contains the node shapes and interpretations in detail:

**Node shape**	**Node description**
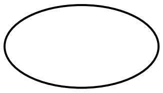	Gene

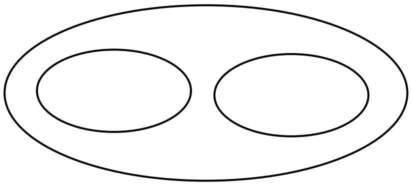	Gene mapped to multiple datasets in the KUPKB or gene found more than one time in a KUPKB dataset
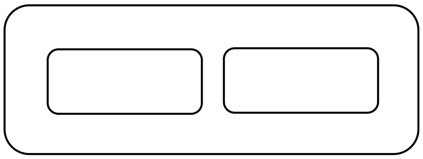	miRNA mapped to multiple datasets in the KUPKB or miRNA found more than one time in a KUPKB dataset
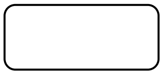	Protein
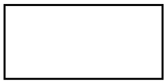	Phosphoprotein
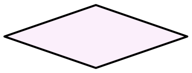	miRNA
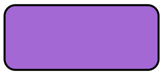	GO cellular component
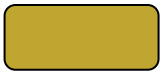	GO molecular function
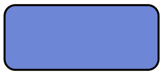	GO biological process
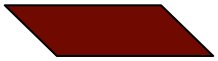	KEGG pathway

Regarding edges, the following image (which is also displayed in the application) depicts the color and edge endings explanations for each edge type.

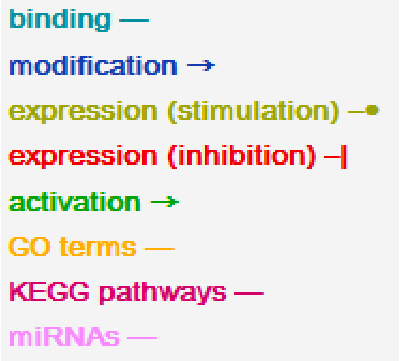

Specifically, the GO edges depict the genes that are hierarchically found under a GO function, the KEGG pathway edges depict the genes that are in the same pathway (the KEGG node) and the miRNA edges are connected to genes that are targets of the miRNA described by a miRNA node.

2.3. Network functionalities

The following sections describe (with examples where possible) several functionalities of the application after the network has been initialized.

2.3.1. 1st and 2nd level neighbors

Supposing that the network has been initialized with the example set of genes/proteins/miRNAs mentioned in section 2.2.1, click on the node named ‘CTGF’ and then with the mouse cursor upon the node, click on the right button of your mouse[1]. The following menu will appear:

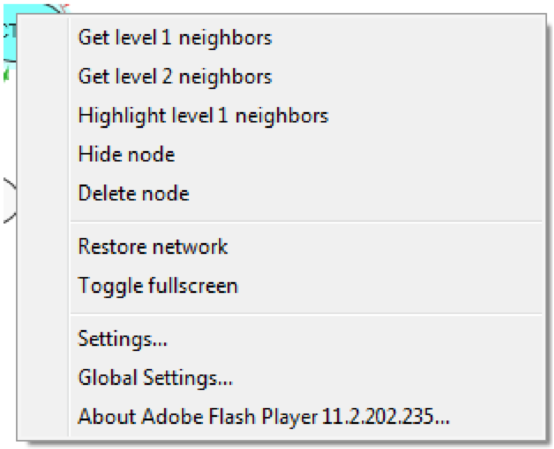

Click on “Get level 1 neighbors”. The application will show a progress window and after a while, the 1st level neighbors (genes/proteins that directly interact with the selected node, with one of the first six interaction types shown in the second figure of section 2.2.2) will appear. The progress window will not disappear immediately as the KUPKB is queried again for kidney locations, diseases and datasets that contain expression data on the new genes which are now added to the network.

[1] The right click does not currently work for Google Chrome in Linux systems. This is a known Google Chrome bug.

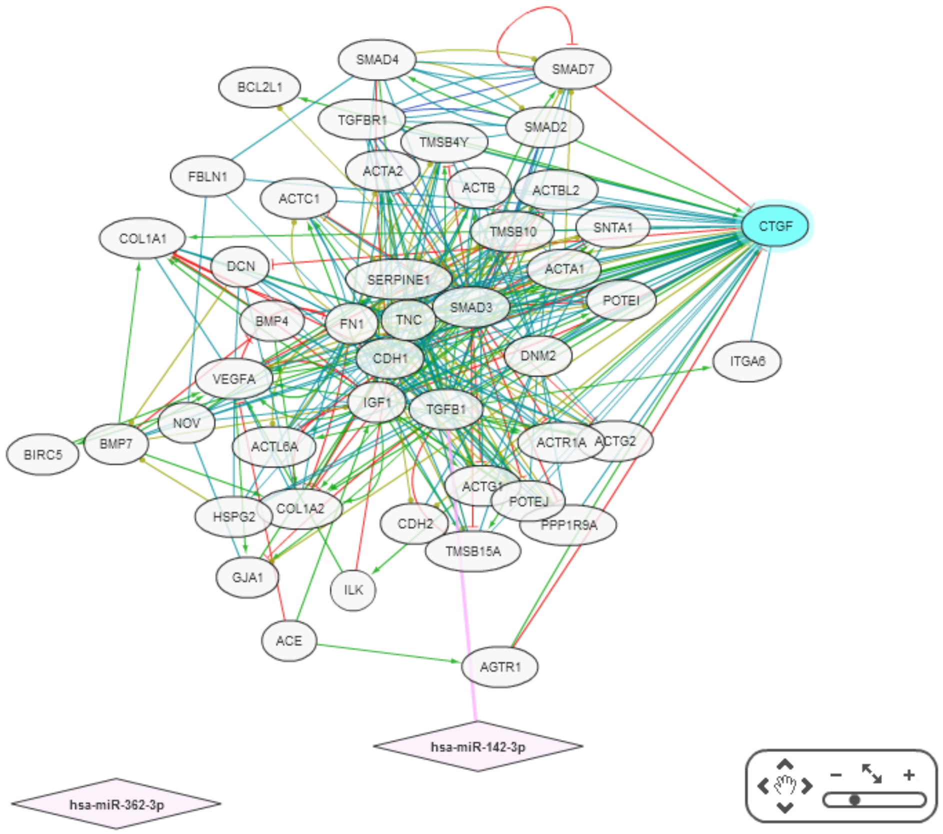

The same actions can be applied for level 2 neighbors. You should keep in mind that when calling for 2nd level neighbors, the application might need a quite large amount of time (depending on the recorded interactions with the selected entity) to process your query. When calling for neighbors, you can also have multiple genes/proteins selected. The application also offers the possibility to highlight the first neighnors of the selected gene/protein by clicking on the “Highlight level 1 neighbors” of the above menu. Neighbors can also be highlighted by visiting the “Advanced” panel and click on the “Mark neighbors” button inside the “Other” panel.

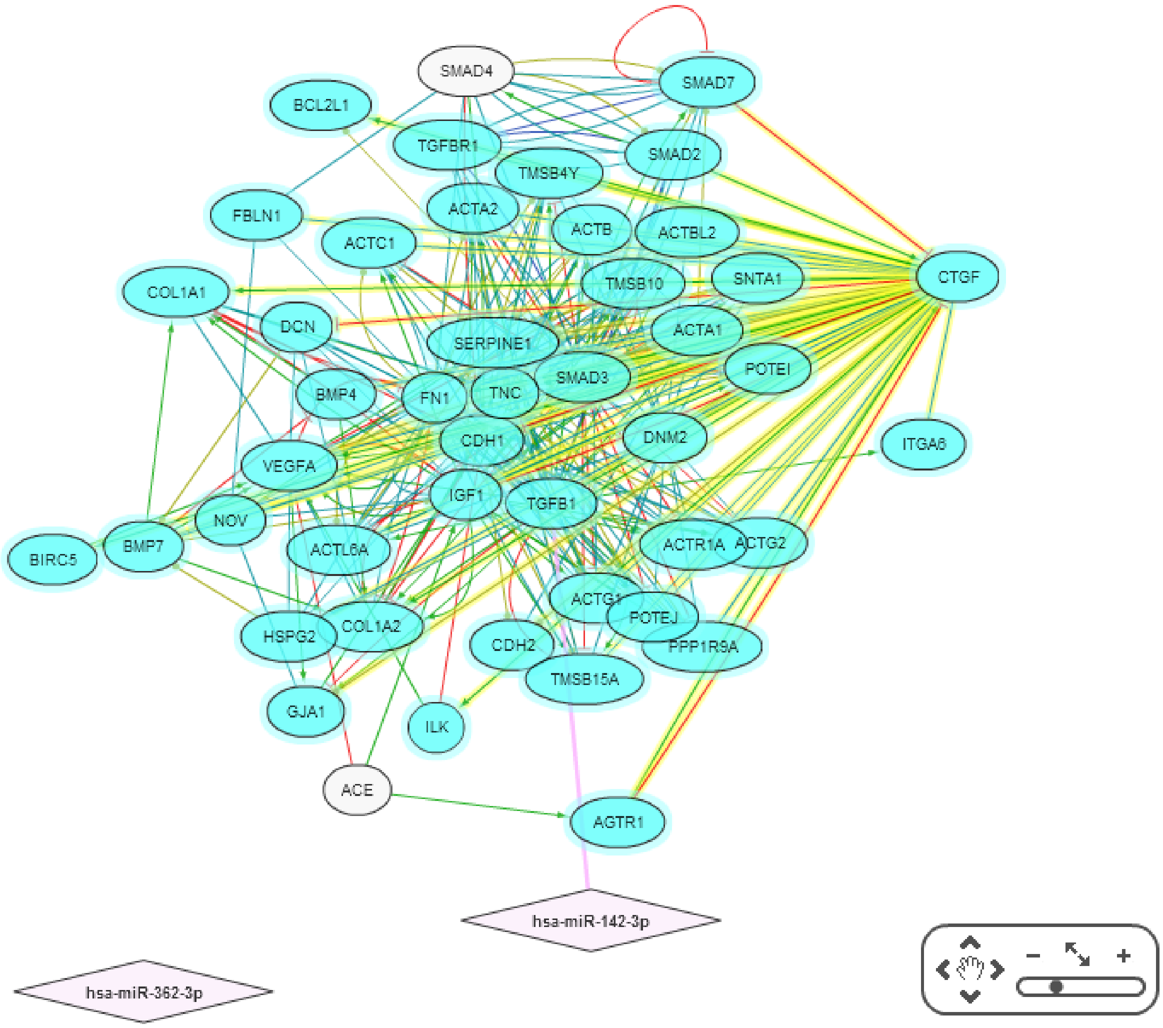

2.3.2. Hiding nodes/edges

To hide one or more nodes from the resulting network, select them either by holding the Shift key while selecting or by clicking inside the network canvas and dragging a square which includes the nodes to be hidden. Then, right click above a node to bring up the menu in the first figure of section 2.3.1 and click "Hide node". It should be noted that a set of hidden nodes is NOT deleted from the network but instead, it becomes invisible and can be restored.

To hide one or more edges from the resulting network, select them either by holding the Shift key while selecting or by clicking inside the network canvas and dragging a square which includes the edges to be hidden. Then, right click above an edge, and the following menu will appear:

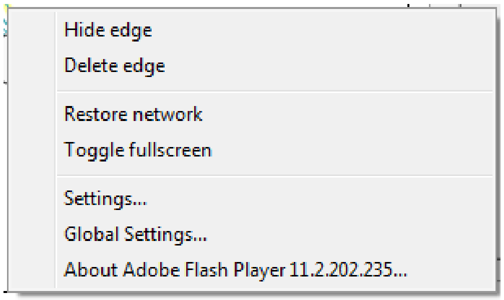

You should then click “Hide edge”. As with hidden nodes, a set of hidden edges is NOT deleted from the network but they become invisible instead and can be restored. Nodes/edges can also be hidden by visiting the “Advanced” tab and clicking on the respective buttons inside the “Other” panel.

2.3.3. Deleting nodes/edges

To delete one or more nodes/edges from the resulting network, select them either by holding the Shift key while selecting or by clicking inside the network canvas and dragging a square which includes the nodes/edges to be deleted. Then, right click above a node/edge to bring up either the menu in the first figure of section 2.3.1 or the menu presented in section 2.3.2 and click “Delete node” or “Delete edge” depending on the item(s) that should be deleted. Nodes/edges can also be deleted by visiting the “Advanced” tab and clicking on the respective buttons inside the “Other” panel. It should be noted that any deleted nodes/edges are PERMANENTLY deleted from the network and CANNOT be restored. If you wish to bring back deleted nodes/edges, you should start over with your search.

2.3.4. Restoring the network

At any time, network elements which are hidden by the process described in section 2.3.2 can be restored, either by bringing up one of the menus described in sections 2.3.1, 2.3.2 and selecting “Restore network”, or by right clicking on an empty area in the network canvas and selecting “Restore network”. Hidden elements can also be restored by visiting the “Advanced” panel and clicking the “Restore network” button inside the “Other” tab.

2.4. Element annotation

On the right side of the network canvas in KUPNetViz, there is a panel that displays additional information regarding a selected network element, entitled “Element information”. This panel can be hidden or made visible again by clicking on “Show element info” which acts as a switch.

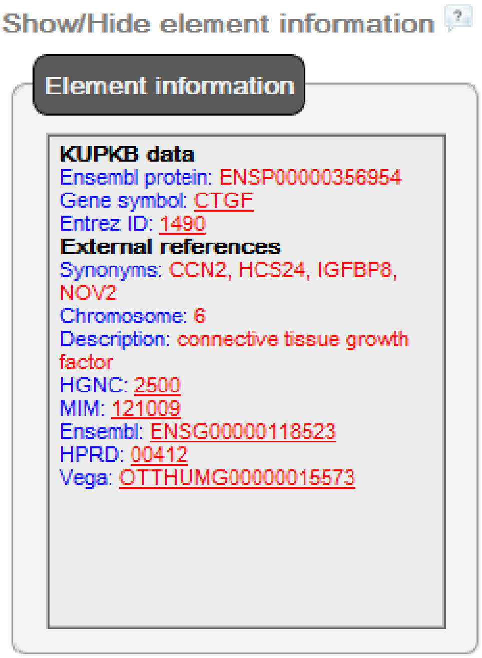

When clicking on a network element (node/edge) in KUPNetViz, the panel “Element information” is filled with several details regarding the selected element. The following sections describe node and edge annotation fields. It should be noted that the number of displayed fields is not standard as when for example a gene has expression information, additional fields regarding its expression strength and/or statistical significance are added.

2.4.1. Node annotation

The following tables describe each dispayed field and its description for nodes. It should be noted that the number of displayed fields is not standard as when for example a gene has expression information, additional field regarding its expression strength and/or statistical significance is added.

KUPKB data

Ensembl protein	The Ensembl accession ID of the protein which is represented by the selected gene/protein
Gene symbol	The HUGO gene symbol of the selected gene/protein
Entrez ID	The Entrez accession number (gene) of the selected gene/protein
Expression strength	The gene/protein expression strength (Weak, Medium, Strong, Absent, Present) of the selected node found in KUPKB datasets (if available).
Differential expression	The gene/protein differential expression characterization (Up, Down, Unmodified) of the selected node found in KUPKB datasets (if available)
Fold change	The gene/protein fold change relative to its control experimental condition found in KUPKB datasets (if available)
p-value	The gene/protein p-value found in KUPKB datasets (if available)
FDR	The gene/protein False Discovery Rate found in KUPKB datasets (if available)
Experiment description	A description of the dataset mapped to the selected node(s). If you move your mouse over the link “here”, a short description will appear in the form of a tooltip on the fly. If you click the link, the description will appear in a separate window.

**External references**	
Synonyms	Gene symbol synonyms found in several databases (source: NCBI)
Chromosome	The chromosome where the selected gene is located
Description	The complete description of the selected gene
HGNC	The HUGO Gene Nomenclature Committee database accession
MIM	The Online Mendelian Inheritance in Man database accession
Ensembl	The Ensembl gene accession
HPRD	The Human Protein Reference Database protein accession
Vega	The Vertebrate Genome Annotation Database accession

**Gene Ontology data**	
GO ID	The Gene Ontolody accession
GO term	The Gene Ontology term annotation
GO category	The Gene Ontology term category (cellular component, biological process, molecular function)

**KEGG pathway data**	
Pathway ID	The KEGG pathway accession
Pathway name	The KEGG pathway name
Pathway class	The KEGG pathway classification

**miRNA data**	
miRNA ID	The miRBase accession
miRNA name	The miRNA name

In most cases of information related to other biological databases, the displayed name is also a link leading to the respective datasheet. If you click on the link, the additional information is displayed in a separate window.

2.4.2. Edge annotation

The following table describes each dispayed field and its description for edges. It should be noted that the number of displayed fields is not standard as when for example a gene has expression information, additional field regarding its expression strength and/or statistical significance is added. In addition, information can be displayed only for one element at a time. If multiple elements are selected, the “Element info” panel simply displays the number and type of selected items.

**Edge data**	
Interaction type	The protein-protein interaction type (one of binding, modification, expression (stimulation), expression (inhibition), activation, GO, KEGG or miRNA connection
Interaction score	The protein-protein interaction score (source: STRING database)
Evidence	Click this link to perform a simple search in PubMed for possible connections between the connected entities

2.5. Adding KUPKB data

Potentially the strongest part of the KUPNetViz is the mapping of KUPKB gene/protein/miRNA expression and statistical significance data to biological interaction networks. The following sections describe analytically the application functionalities regarding its interaction with the KUPKB which is performed using the 2nd tab of the application ("Add KUPKB data").

2.5.1. The KUPKB gene/protein data panel

This panel displays KUPKB entries regarding the kidney diseases, locations and published studies which contain or are referred to at least one of the gene/protein nodes present in the network which was created after quering for genes/proteins/miRNAs using the first tab of the application.

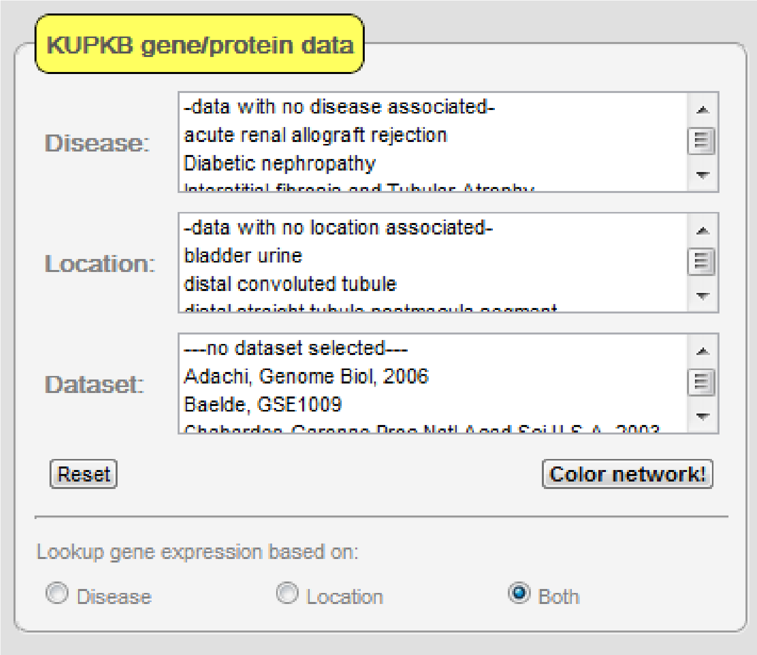

The contents are dynamic, meaning that they are refilled each time a new network is created or new nodes are added (e.g. in the forms of a gene's neighbors). They are also free or restricted according to the choice of the field "Lookup gene expression based on:" or the search modes in the "Search options" panel of the "Advanced tab", which is explained in section 2.7.1. The disease, location and dataset lists allow either single or multiple entry selections by pressing the Ctrl key while making selections. Because in general, multiple selections may lead to readability, comprehensibility and visualization complexity problems, you can disable this feature by unchecking the respective checkboxes in the "Coloring options" panel of the "Advanced tab". The "Dataset" list is repopulated each time a selection is made in the "Disease" and "Location" lists. The following table describes the behavior of the repopulation according to the selection of "Lookup gene expression based on:"

**Option**	**Behavior**
Disease	The datasets containing gene/protein expression are fetched from KUPKB based only on the selected

	disease(s), regardless the kidney location. The "Location" list is disabled.
Location	The datasets containing gene/protein expression are fetched from KUPKB based only on the selected location(s), regardless the disease. The "Disease" list is disabled.
Both	The datasets containing gene/protein expression are fetched from KUPKB based on both the selected disease(s) and location(s). Both lists are enabled and the search becomes stricter.

At any time, the contents of all the lists can be reverted (as long as there is no change in the network, where in this case the lists are re-initialized according to the new queried entities), to their initial ones by pressing the "Reset" button. The "Reset" button also clears any gene/protein expression (node coloring) mapping present in the network. The "Color network!" button will map gene/protein expression data found in the KUPKB based on the selections made in all 3 lists and one or several nodes will be colored according to the legend on the right side of the network canvas.

As an example, based on the network created with the entities of section 2.2.1, go to the "Add KUPKB data" tab and in the "KUPKB gene/protein data" panel, select "acute renal allograft rejection" in the "Disease" list. The "Location" list will be repopulated and the new contents will be "bladder urine" and "kidney". Select "kidney" in the "Location" lists. The "Dataset" list will now contain only "Head, GSE1563". Select this dataset and click on the "Color network!" button. The network will now look like the following figure:

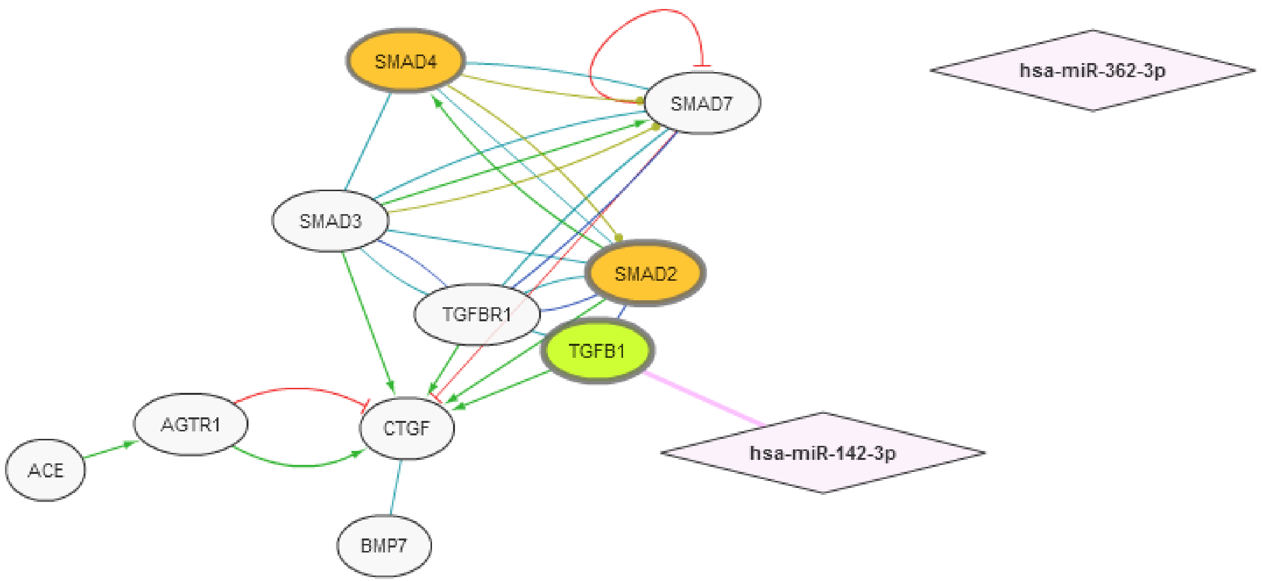

Click on TGFB1. The "Element info" panel will be filled with information regarding TGFB1 retrieved from the KUPKB dataset "Head, GSE1563".

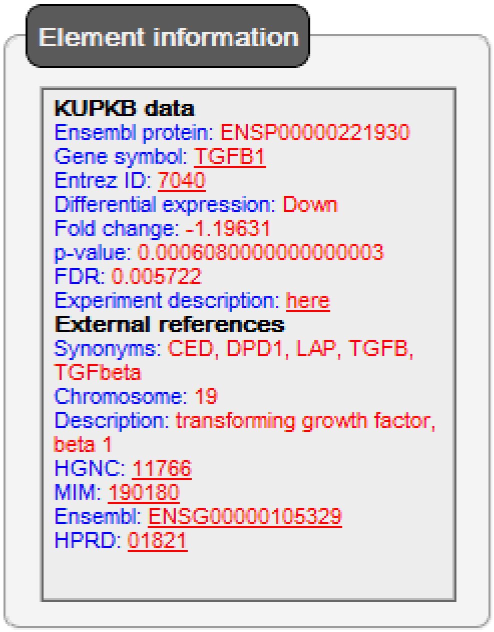

Move your mouse over the “here” link next to the “Experiment description” field. A short description of the “Head, GSE1563” dataset will appear:

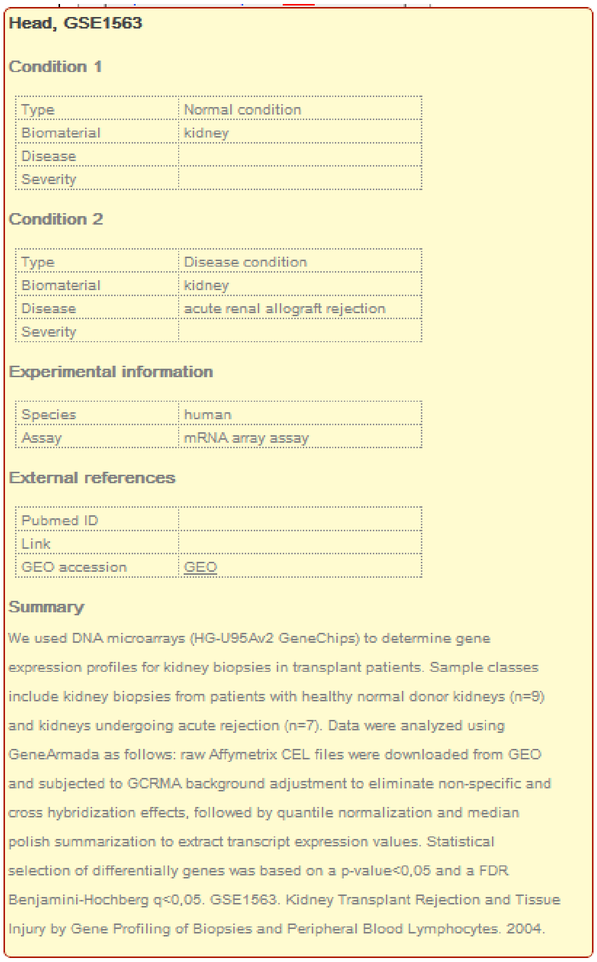

Head, GSE1563

Condition 1

Type	Normal condition
Biomaterial	kidney
Disease	
Severity	

Condition 2

Type	Disease condition
Biomaterial	kidney
Disease	acute renal allograft rejection
Severity	

Experimental information

Species	human
Assay	mRNA array assay

External references

Pubmed ID	
Link	
GEO accession	GEO

Summary

We used DNA microarrays (HG-U95Av2 GeneChips) to determine gene expression profiles for kidney biopsies in transplant patients. Sample classes include kidney biopsies from patients with healthy normal donor kidneys (n=9) and kidneys undergoing acute rejection (n=7). Data were analyzed using GeneArmada as follows: raw Affymetrix CEL files were downloaded from GEO and subjected to GCRMA background adjustment to eliminate non-specific and cross hybridization effects, followed by quantile normalization and median polish summarization to extract transcript expression values. Statistical selection of differentially genes was based on a p-value<0,05 and a FDR Benjamini-Hochberg q<0,05. GSE1563. Kidney Transplant Rejection and Tissue Injury by Gene Profiling of Biopsies and Peripheral Blood Lymphocytes. 2004.

You can also display multiple datasets at once. For this, in the “KUPKB gene/protein data” panel, click the “Reset” button and this time (assuming all default selections), select “acute renal allograft rejection” and “Diabetic nephropathy” in the “Disease” list. The “Location” list will be repopulated and the new contents will be “bladder urine” and “kidney”. Select “kidney” in the “Location” lists. The “Dataset” list will now contain “Head, GSE1563” and “Ronney, FEBS Lett, 2011”. Select both datasets and click on the “Color network!” button. The network will now look like the following figure:

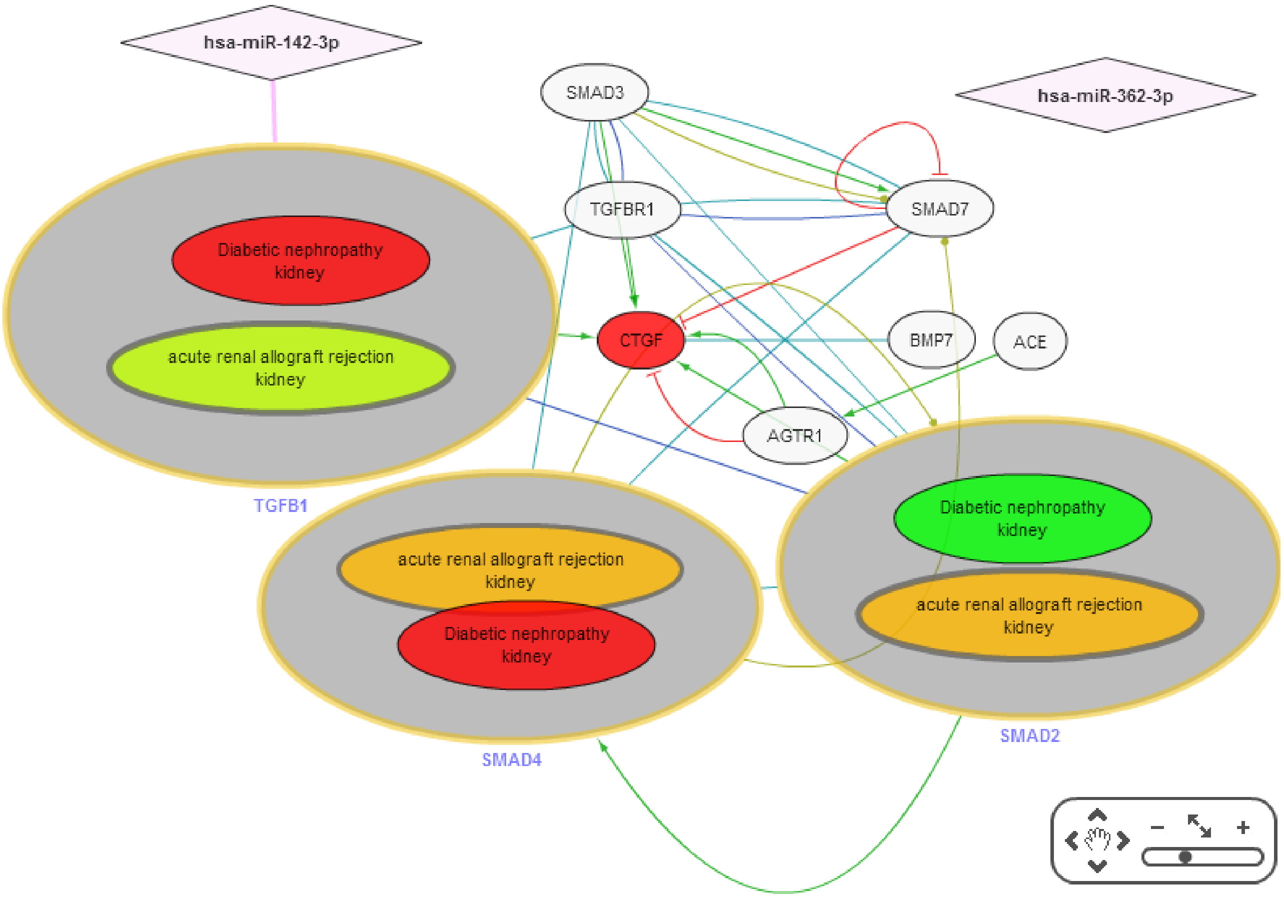

You now notice that there are compound nodes in the network. In this case, it means that TGFB1, SMAD4 and SMAD2 were found in both selected datasets, with different expression values. The internal node annotations (e.g. “Diabetic nephropathy” + “kidney”) depict the dataset and the conditions which are mapped to this internal node (and also indicate the dataset).

Click on the red internal node of TGFB1. The “Element info” panel will now be filled with information regarding TGFB1 retrieved from the KUPKB dataset “Rooney, FEBS Lett, 2011”. If you click on the green internal node of TGFB1, the “Element info” panel will be filled with information regarding TGFB1 retrieved from the KUPKB dataset “Head, FEBS Lett, 2011”.

2.5.2. The KUPKB miRNA data panel

This panel displays KUPKB entries regarding the kidney diseases, locations and published studies which contain or are referred to at least one of the miRNA nodes present in the network which was created after quering for genes/proteins/miRNAs using the first tab of the application.

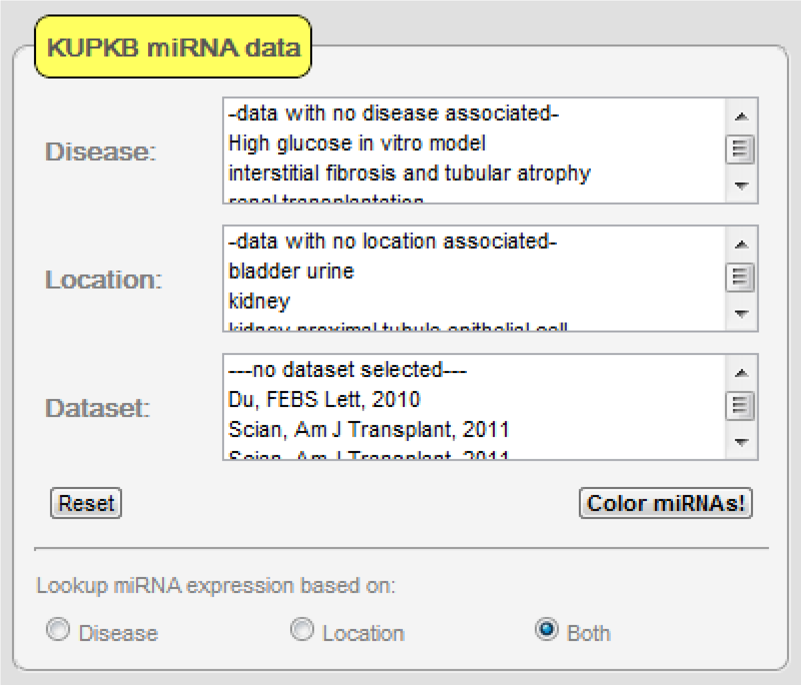

The lists behavior is the same as described in section 2.5.1. The following table describes the behavior of the repopulation according to the selection of “Lookup miRNA expression based on:”

**Option**	**Behavior**
Disease	The datasets containing miRNA expression are fetched from KUPKB based only on the selected disease(s), regardless the kidney location. The “Location” list is disabled.
Location	The datasets containing miRNA expression are fetched from KUPKB based only on the selected location(s), regardless the disease. The “Disease” list is disabled.
Both	The datasets containing miRNA expression are fetched from KUPKB based on both the selected disease(s) and location(s). Both lists are enabled and the search becomes stricter.

The “Reset” and “Color miRNAs!” buttons work as in section 2.5.1 but for miRNAs. As an example, based on the network created with the entities of section 2.2.1, go to the “Add KUPKB data” tab and in the “KUPKB miRNA data” panel, select “interstitial fibrosis and tubular atrophy” and “renal transplantation” in the “Disease” list and “kidney” in the “Location” lists. The “Dataset” list will now contain only “Scian, Am J Transplant, 2011”. Select this dataset and click on the “Color miRNAs!” button. The network will now look like the following figure:

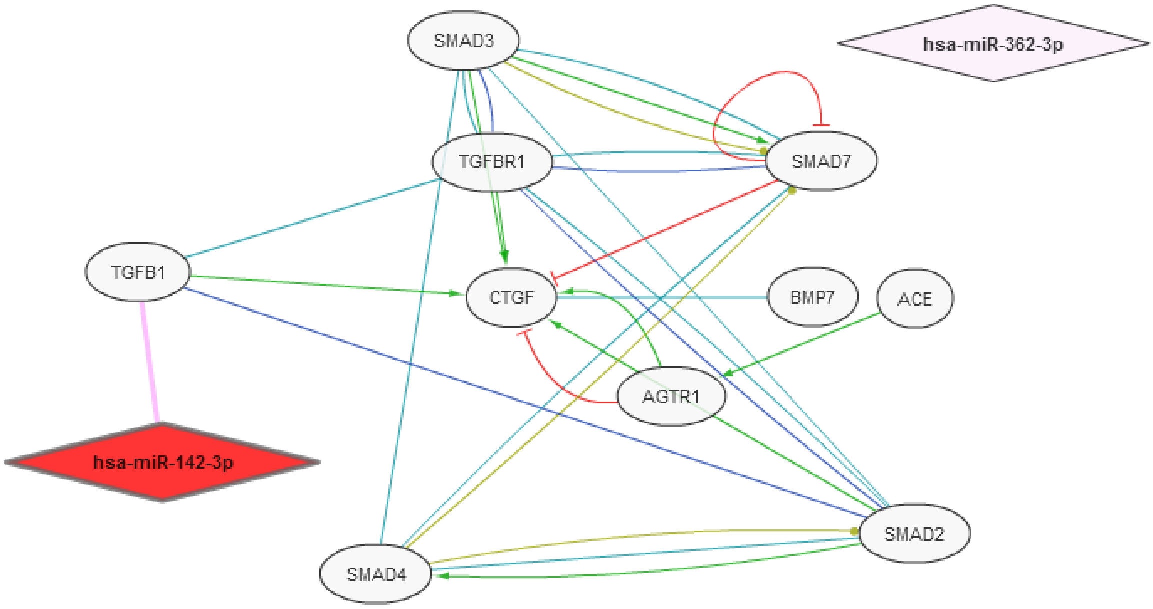

Regarding the selection of multiple dataset and the case of compound miRNA nodes, similar things apply as in section 2.5.1.

2.5.3. The Displayed interactions panel

From this panel, you can control what types of interactions are displayed in the network. For example, you can hide binding and display only expression and activation interactions. The color of the font next to the checkboxes reflects the color of the edges depicting the respective interactions in the network. As an example, using the network initialized in section 2.2.1, uncheck the binding and modifications. The network should be similar to the following figure.

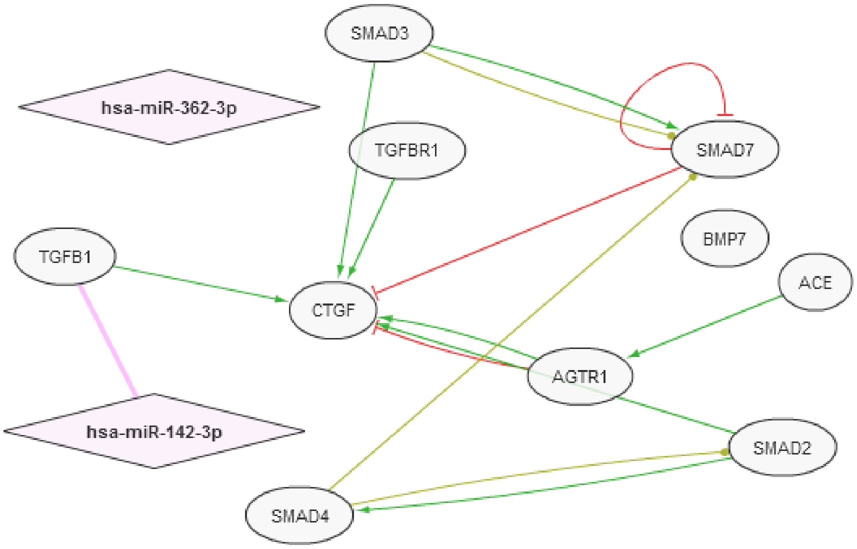

2.5.4. Displaying neighbors

This functionality has been described in section 2.3.1. It can additionally be performed using the respective buttons in the "Other" panel of the "Add KUPKB data" tab. The "Toggle network fullscreen" will maximize the top part of the application containing the network canvas.

2.6. Adding functional and pathway data

Another important part of the KUPNetViz is the mapping of KUPKB gene/protein elements to Gene Ontology terms that describe them, KEGG pathways that contain them and miRNAs that target them. The following sections describe analytically the above functionalities which are performed using the 3rd tab of the application ("Add GO, KEGG or miRNA data").

2.6.1. The Gene Ontology panel

The Gene Ontology (GO) panel contains a list with the GO terms which were found to describe at least one of the network genes/proteins. Above the list, three buttons control the GO category (Cellular Component, Molecular Function, Biological Process) and clicking on them fills the list accordingly. The list is filled upon network initialization and is repopulated when new network elements (e.g. a gene's neighbors) are added in order to contain possible additional GO terms.

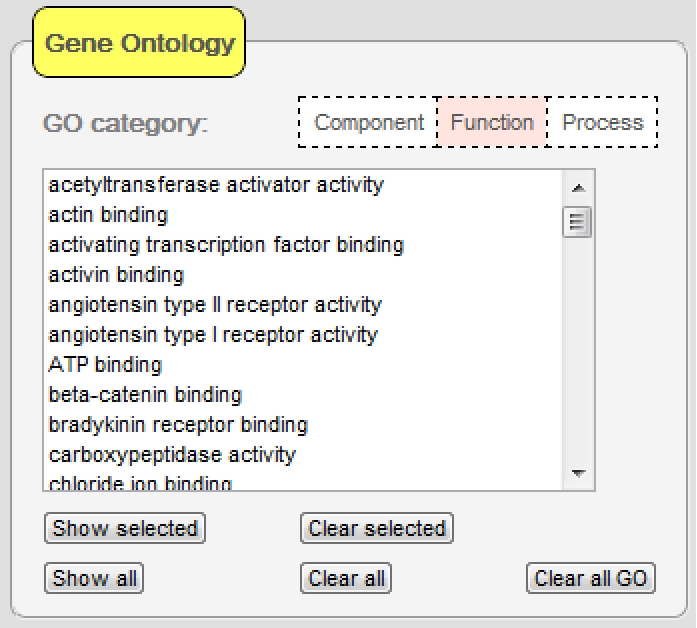

In order to display a GO term and its connections to genes/proteins in the network, select one or more terms from the category of interest and click "Show selected". Alternatively, you can double-click on a single term to add it to the network. The following table describes the functions of the buttons below the list:

**Button**	**Function**
Show selected	Creates GO nodes and edges to genes/proteins for the selected item(s) of the current category.
Clear selected	Removes selected GO node(s) from the network.

Show all	Creates GO nodes and edges to genes/proteins for all items in the GO list and current category (*it may cause display, readability, comprehensibility and speed problems*).
Clear all	Removes all GO nodes of the *current* category from the network.
Clear all GO	Removes *all* GO terms (regardless of category) from the network.

As an example, using the network initialized in section 2.2.1, go to the “Add GO, KEGG or miRNA data” and on the “Gene Ontology” panel, select “nucleolus” and click “Show selected”. Next, click on the “Function” button and on the list that appears and double-click on “ATP binding”. Last, click the “Process” button above the list and double-click “induction of apoptosis”. The network should now look like the following figure:

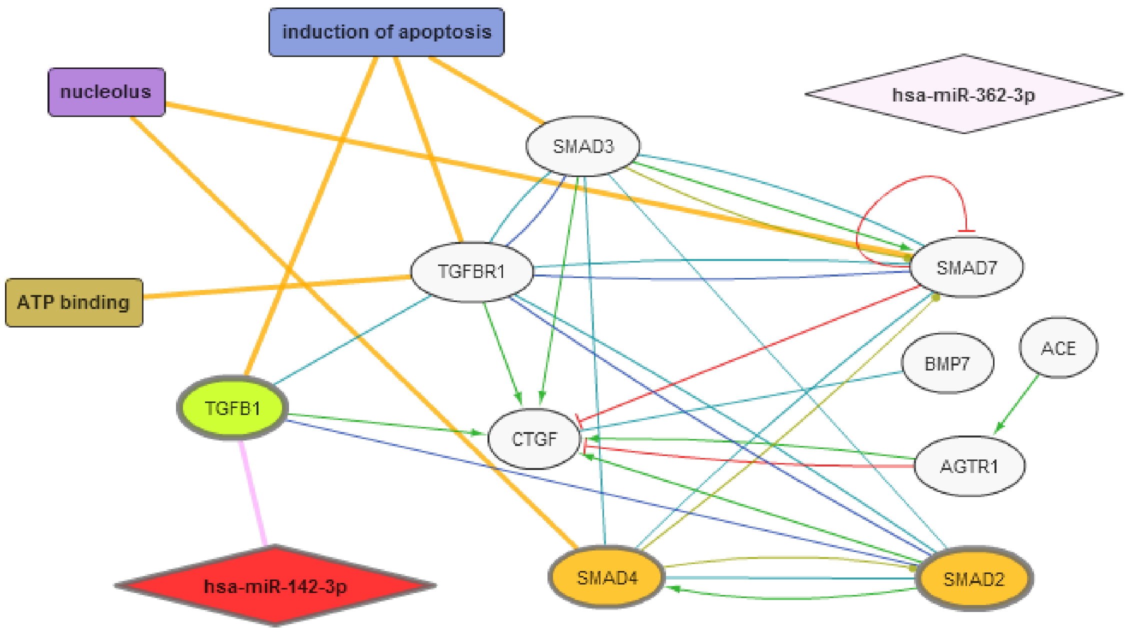

2.6.2. The KEGG pathways panel

The KEGG pathways panel contains a list with the KEGG pathways which were found to contain at least one of the network genes/proteins. The list is filled upon network initialization and is repopulated when new network elements (e.g. a gene’s neighbors) are added in order to contain possible additional KEGG pathways. KEGG pathways are categorized inside the pathway list according to their KEGG classification (bold unselectable elements).

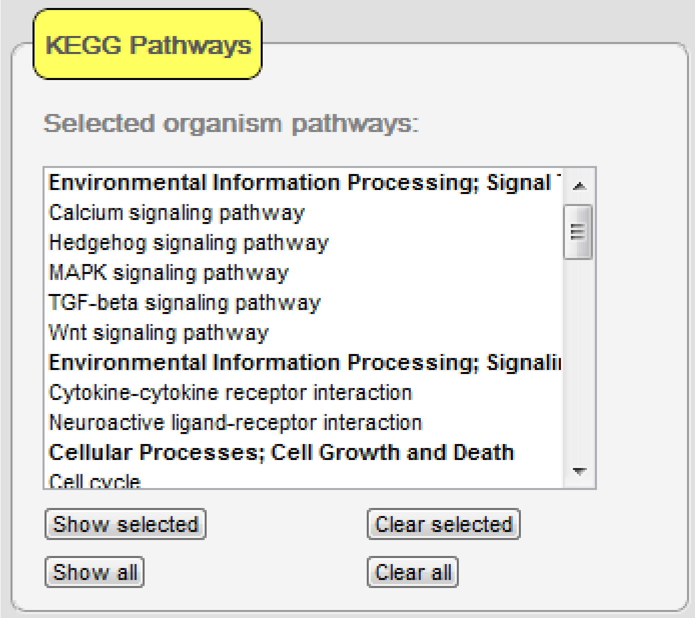

In order to display a KEGG pathway and its connections to genes/proteins in the network, select one or more pathways and click "Show selected". Alternatively, you can double-click on a single item to add it to the network. The following table describes the functions of the buttons below the list:

**Button**	**Function**
Show selected	Creates KEGG nodes and edges to genes/proteins for the selected item(s).
Clear selected	Removes selected KEGG node(s) from the network.
Show all	Creates KEGG nodes and edges to genes/proteins for all items in the KEGG list (*it may cause display, readability, comprehensibility and speed problems*).
Clear all	Removes all KEGG nodes from the network.

As an example, using the resulting network of section 2.6.1, go to the "Add GO, KEGG or miRNA data" and on the "KEGG Pathways" panel, select "MAPK signaling pathway" and click "Show selected". The network should now look like the following figure:

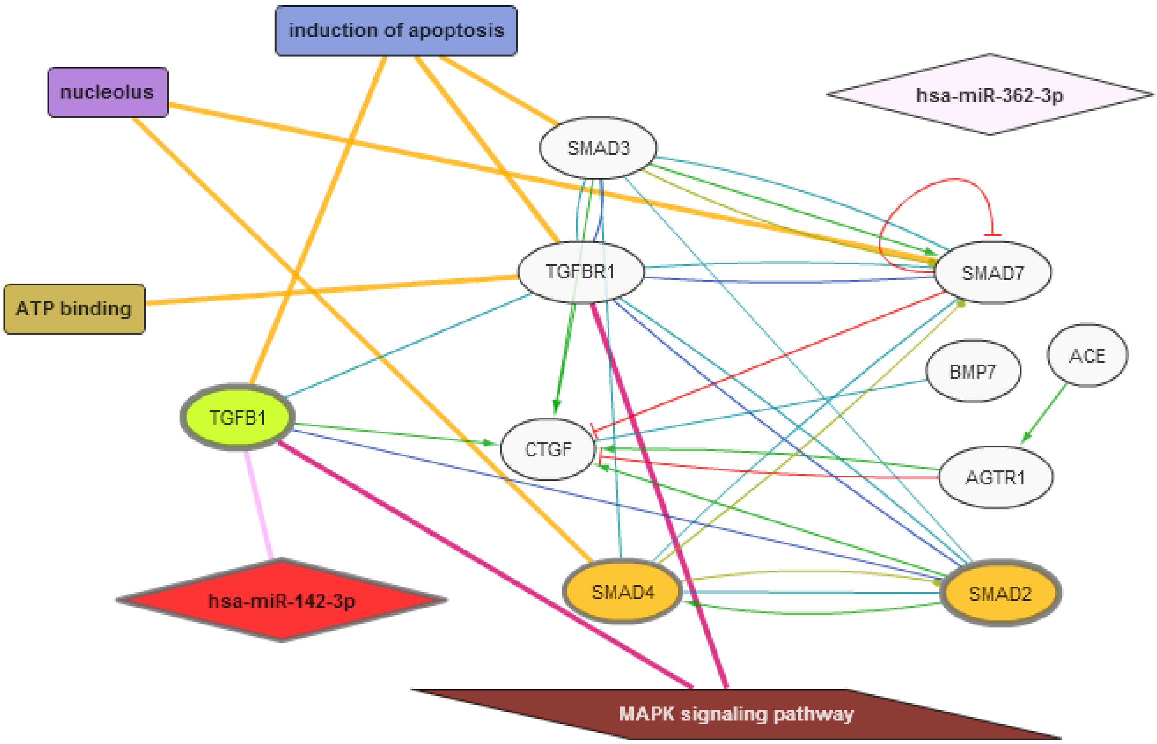

2.6.3. The miRBase microRNA panel

The miRBase pathways panel contains a list with the miRNAs which were found to target at least one of the network genes/proteins. The list is filled upon network initialization and is repopulated when new network elements (e.g. a gene's neighbors) are added in order to contain possible additional miRNAs.

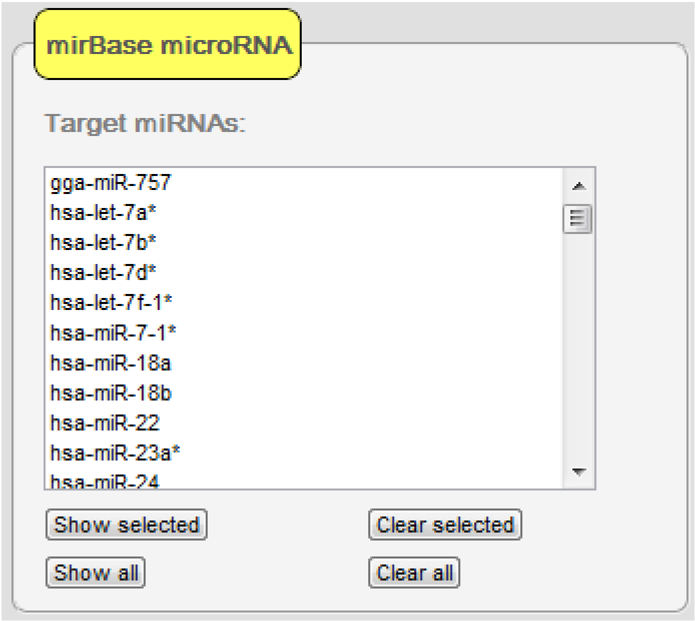

In order to display a miRNA element and its connections to genes/proteins in the network, select one or more miRNAs and click “Show selected”. Alternatively, you can double-click on a single item to add it to the network. The following table describes the functions of the buttons below the list:

**Button**	**Function**
Show selected	Creates miRNA nodes and edges to genes/proteins for the selected item(s).
Clear selected	Removes selected miRNA node(s) from the network.
Show all	Creates miRNA nodes and edges to genes/proteins for all items in the miRNA list (*it may cause display, readability, comprehensibility and speed problems*).
Clear all	Removes all miRNA nodes from the network.

As an example, using the resulting network of section 2.6.2, go to the “Add GO, KEGG or miRNA data” and on the “mirBase microRNA” panel, select “has-miR-744” and click “Show selected”. The network should now look like the following figure:

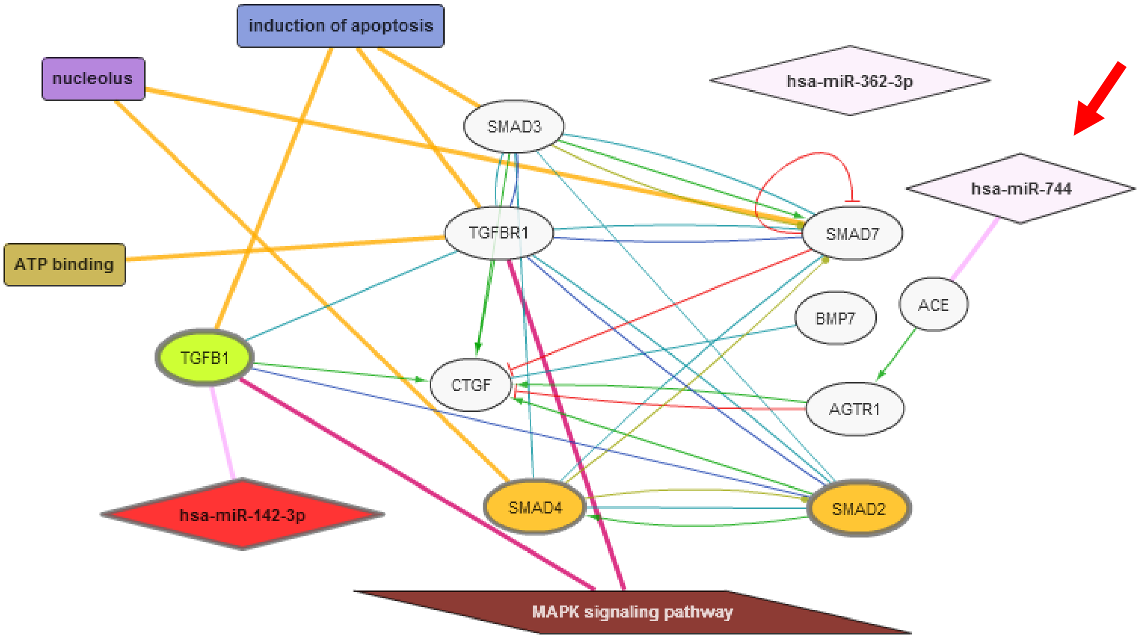

2.7. Advanced options

Apart from the basic functionalities described in the previous sections, KUPNetViz has a set of advanced functionalities, controlling mostly several search, coloring and display options. The following sections describe these functionalities with examples where possible.

2.7.1. The Search options panel

The Search options panel contains several options regarding the method that the KUPKB data (gene/protein/miRNA) lists are populated in the 2nd application tab “Add KUPKB data”, the neighbor search

mode, the edges-relationships that are displayed among nodes and the interactions threshold for an interacting gene/protein to be considered as “neighbor” (explanations on this score can be found in the STRING protein-protein interaction database, http://www.string-db.org.

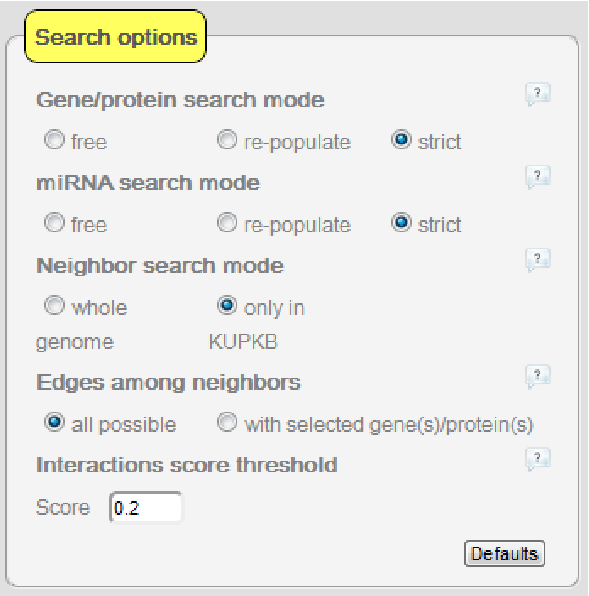

The following table describes the available options in the “Search options” panel. At any time, pressing the “Defaults” button will restore all the original options.

Gene/protein search mode

free	Free search displays all the diseases/locations resulting from the queried genes/proteins constantly and only datasets are repopulated. With this type of search, while all the found kidney locations and diseases are displayed all the time, the selection of a location, disease or a combination of the two (see section 2.5.1) does not guarantee an existing dataset fulfilling the criteria and thus, no expression mapping for the network.
re-populate	Re-populate refills the disease/location lists upon a selection in either of them. For example, if you select “acute renal allograft rejection” in the disease list, the location list will be re-populated based on locations which have been associated with this pathology in KUPKB datasets. If you select “kidney” in the location list, the disease list will be re-populated based on diseases which have been associated with this locations in KUPKB datasets. This functionality is similar to the “Disease” or “Location” options described in section 2.5.1 (field “Lookup gene expression based on:”) but with a more dynamic view.
strict	Strict repopulates only once. In this way, the list into which the first selection is made is “locked” and the other one is repopulated based on any selection of the first. For example, if you select “acute renal

allograft rejection" in the disease list, the location list will be re-populated based on locations which have been associated with this pathology in KUPKB datasets. Any selection in the location list will not cause a repopulation of the disease list. However, if you wish to completely change the expression search criteria, you will have to press the "Reset" button.

miRNA search mode

free	Same as the "Genes/proteins search mode" "free" option but for miRNAs.
re-populate	Same as the "Genes/proteins search mode" "re-populate" option but for miRNAs.
strict	Same as the "Genes/proteins search mode" "strict" option but for miRNAs.

Neighbor search mode

whole genome	When calling for neighbours, if this option is checked, the application searches for neigboring genes/proteins in the whole genome of the selected organism in the 1st panel of the application.
only in KUPKB	When calling for neighbours, if this option is checked, the application searches for neigboring genes/proteins only present in KUPKB datasets.

Edges among neighbours

all possible	When calling for neighbours, if this option is checked, the application displays all possible relationships also among the new nodes added to the network.
with selected gene(s)/protein(s)	When calling for neighbours, if this option is checked, the application displays only the relationships with the node(s) that was/were selected when calling for neighbours.

Interactions score threshold

score	The interaction score threshold from STRING database.

2.7.2. The Coloring options panel

The Coloring options panel contains several options regarding the gene/protein/miRNA node coloring as well whether multiple selections are allowed in the disease, location and dataset lists.

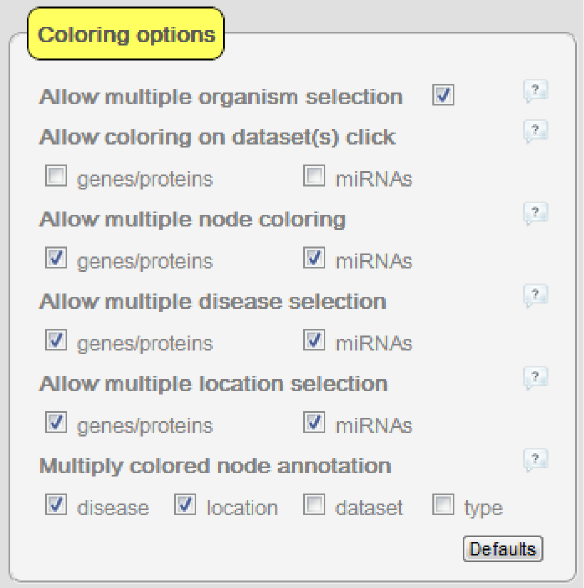

The following table describes the available options in the “Search options” panel. At any time, pressing the “Defaults” button will restore all the original options.

Allow multiple organism selection
Check this box to allow multiple selections to be made in the organism list in the 1st tab of the application in order to search the queried genes/proteins/miRNAs in the selected organisms. You should be careful when using this option though as the resulting view might be extremely fuzzy and slow down the application.

Allow coloring on dataset(s) click

genes/proteins	Check this box to allow the coloring of gene/protein nodes simply by clicking on the dataset name(s) in the list. Otherwise, the “Color network!” button must be pressed. Uncheck this box to explore the datasets without excessive workload for the server or check it for fast exploration of smaller queries.
miRNAs	Check this box to allow the coloring of miRNA nodes simply by clicking on the dataset name(s) in the list. Otherwise, the “Color miRNAs!” button must be pressed. Uncheck this box to explore the datasets without excessive workload for the server or check it for fast exploration of smaller queries.

Allow multiple node coloring

genes/proteins	Check this box to allow mutliple colors in a gene/protein node in the network, if found to be present in multiple selected datasets. Expression in multiple datasets will be represented by a larger node containing several smaller ones (see section 2.2.2), according to the number of the datasets. Each one will be colored based on the expression recorded in the respective dataset. If unchecked, a grey node will represent multiple found genes.
miRNAs	Check this box to allow mutliple colors in a miRNA node in the network, if found to be present in multiple selected datasets. Expression in multiple datasets will be represented by a larger node containing several smaller ones (see section 2.2.2), according to the number of the datasets. Each one will be colored based on the expression recorded in the respective dataset. If unchecked, a grey node will represent multiple found miRNAs.

Allow multiple disease selection

genes/proteins	Check this box to allow multiple selections to be made in the disease lists connected to the queried genes/proteins in the 2nd tab of the application. You should be careful when using this option though as the resulting view might be fuzzy and slow down the application.
miRNAs	Check these boxes to allow multiple selections to be made in the disease lists connected to the queried miRNAs in the 2nd tab of the application. You should be careful when using this option though as the resulting view might be fuzzy and slow down the application.

Allow multiple location selection

genes/proteins	Check this box to allow multiple selections to be made in the location lists connected to the queried genes/proteins in the 2nd tab of the application. You should be careful when using this option though as the resulting view might be fuzzy and slow down the application.
miRNAs	Check these boxes to allow multiple selections to be made in the location lists connected to the queried miRNAs in the 2nd tab of the application. You should be careful when using this option though as the resulting view might be fuzzy and slow down the application.

Multiple colored node annotation

disease<br>location<br>dataset<br>type	Check these boxes to control the information displayed inside nodes when multiple node coloring is allowed. Having everything checked might render the network too complex to view when querying many genes/proteins. Type is either gene or protein.

2.7.3. Other advanced options

The Coloring options panel contains several miscellaneous options controlling node and edge labels, redundant node and edge controls and most importantly, the options that control the appearance of nodes that represent genes/proteins when multiple species are selected and the respective edges.

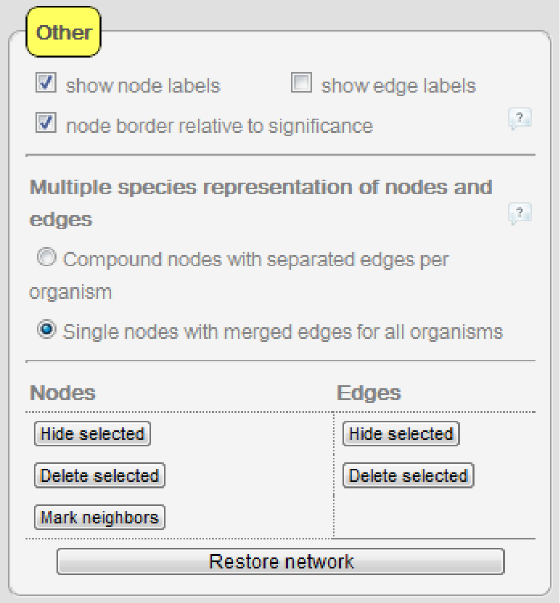

The following table describes the miscellaneous options at the top of the panel:

**Miscellaneous options**	
show node labels	Check this box to show/hide node labels (shown by default).
show edge labels	Check this box to show/hide edge labels (hidden by default).
node border relative to significance	Check this box to make the thickness of a node's border relative to its statistical significance (p-value) when a node is mapped to a KUPKB dataset and when there is a p-value for that gene in the selected dataset(s).

The "Multiple species representation of nodes and edges" field is explained in detail in section 3.7. The "Nodes" and "Edges" fields contain buttons regarding the visibility and deletion of nodes and edges as well as the network restoration which are explained in sections 2.3.2 – 2.3.4.

3. Using the KUPNetViz in multiple species mode

One of the strongest parts in KUPNetViz is the ability to query for your favorite molecules in multiple species. When working with multiple species and depending on the chosen way of gene/protein representation, relationships and gene/protein expression can be extrapolated from one organism to another. While this assumption may not be entirely biologically accurate, it allows the easy formulation of such extrapolation hypotheses. The researcher can then judge the fundamental validity of these hypotheses and proceed either by rejecting a hypothesis and formulate another, or by creating a list of plausible hypothesis which can ultimately be verified in the lab.

3.1. Searching for genes/proteins/miRNAs

The process is the same as the one described in section 2.1. Only this time, you have to select multiple species in the species list of the 1st tab of the application ("Molecule search") by holding the Ctrl key while selecting organisms. Be sure that the "Allow multiple organism selection" option is checked in the "Coloring options" panel of the "Advanced" tab.

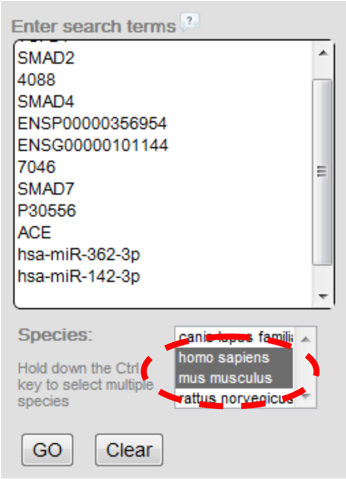

3.2. Network description

3.2.1. Network initialization

The network is initialized exactly with the same way as described in section 2.2.1 with the only difference being the multiple species.

3.2.2. Node and edge description

Nodes and edges are similar to the ones described in section 2.2.2. Only this time, when "Single nodes with merged edges for all organisms" is checked in the "Other" panel of the "Advanced" tab, nodes are

represented by circles with the name of the gene below them, in bold lowercase letters. Edge colors and types are the same, only this time thicker to reflect the multiple species selection.

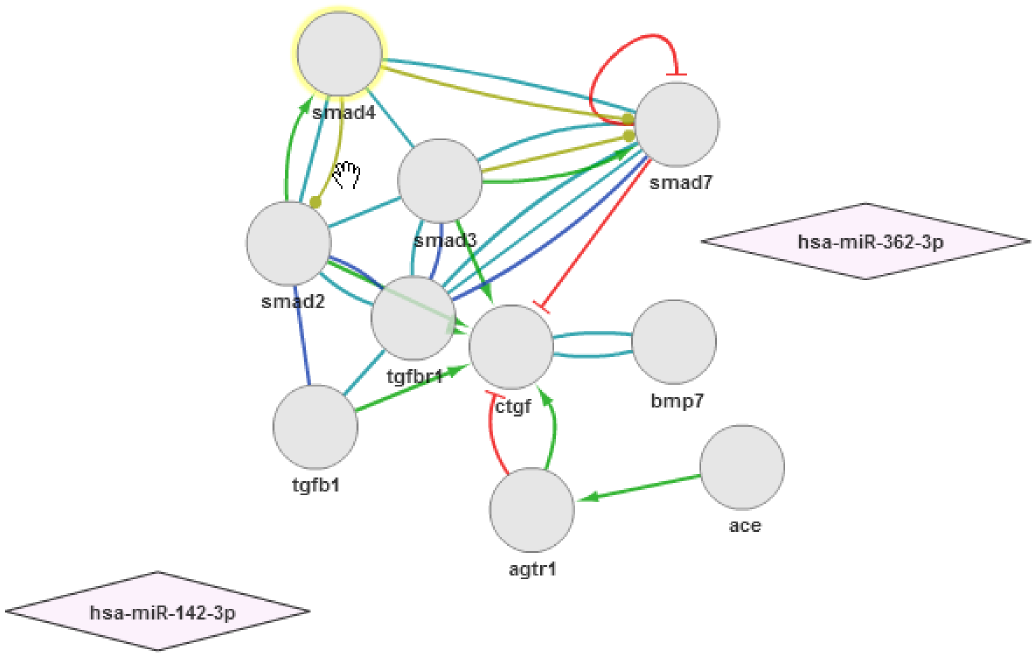

3.3. Network functionalities

The network functionalities remain the same as the ones described in section 2, only this time, genes and edges correspond to supergenes and superedges respectively.

3.4. Element annotation

The element annotation is the same as described in section 2.4. You should keep in mind that for genes representing homologues from multiple organisms ("supergenes"), only the gene symbol is presented as an annotation element with a link to the GeneCards database.

3.5. Adding KUPKB data

The process of adding KUPKB data in a multispecies network is the same as the one described in section 2.5. The only difference is that disease, location and dataset lists now will contain more entries as a result of the multiple organism selection.

3.6. Adding functional and pathway data

The process of adding Gene Ontology, KEGG pathway and miRNA nodes to the network is the same as the one described in section 2.6, with the only difference the KEGG pathways. In the case of multiple organisms, the KEGG pathways list does not contain anymore organism specific pathways but the KEGG reference (“map”, see KEGG website for further details) pathways. The connections are based on the reference pathways so it is possible that in many cases, they are not as complete as the organism specific ones. When “Compound nodes with separated edges per organism” is selected in the “Other” panel in the “Advanced” tab of the application, KEGG pathways become organism specific.

3.7. Advanced options

The advanced options are the same as in single species mode and described in section 2.7. The only difference is that in this case the options in the “Multiple species representation of nodes and edges” become functional and are explained in the table below:

**Multiple species representation of nodes and edges**	
Compound nodes with separated edges per organism	In this case, the nodes representing genes/proteins in multiple organisms are displayed as grey circles with the name of the entity below them (“supergenes”). Organism specific nodes are hidden under this compound node and the edges representing protein-protein interactions have the same colour but they are thicker to indicate the multiple species mode. When one or more datasets are selected from the KUPKB gene/protein data list and the “Color network” button is pressed, the “supergenes” are internally colored with two possible ways: i) if the gene/protein is expressed only once in the selected dataset(s), a colored single-species node appears inside the “supergene”, and ii) if the gene/protein is expressed more than once in the selected dataset(s), a compound node containing dataset annotations (described in section 2.5.1, see the last figure of the section and the explanation) appears inside the “supergene”. A combination of the above is possible, depending on the number of datasets selected and their mapping to the current network. If multiple node coloring for genes/proteins is not allowed (see section 2.7.2), the “supergene” becomes grey with an orange outline (like the respective coloring in single species mode).
Single nodes with merged edges for all organisms	In this case, all the representations are the same as in single-species mode, but this time, gene/protein nodes are encompassed by a “supergene” node and all interactions become species specific and there is no extrapolation.

4. View and Export

This section describes briefly some advanced options regarding the network layout algorithms (network "views") and the basic image and text formats that the network can be exported to. All these options are available through a menu displayed on the top side of the application.

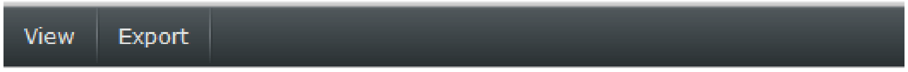

4.1. View

There are 5 view or layout algorithms in KUPNetViz which are the layout algorithms supported by the Cytoscape Web library (http://cytoscapeweb.cytoscape.org/). These layouts are the Force Directed layout (the layout that is applied when "Default" and "Help! Many genes!" items are clicked), the Circle layout, the Tree layout, the Radial layout and the Compound Spring Embedder layout (the layout that is applied when the "Compound" item is clicked).

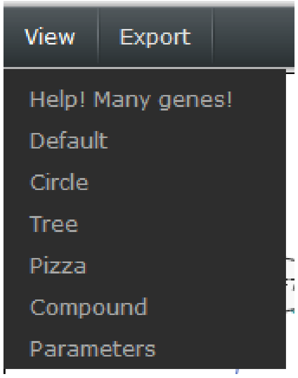

The Compound item can be used only when compound nodes (multi-species mode or multiply colored nodes) are present on the network. All the other layouts are available only when the network does not contain any compound nodes. The Parameters item will display a dialog where the user can change the parameters that are used by each layout algorithm ti properly display the network. The description of these parameters is outside the scope of this guide, but the interested user can refer to the Cytoscape Web website (http://cytoscapeweb.cytoscape.org/).

4.2. Export

The network can be exported either in high quality image formats (PDF, PNG and SVG formats) to be displayed outside the application, or in text formats (SIF, GRAPHML, XGMML) that can be used to import the network in other graph analysis applications. This can be done through the "Export→Image" or through the "Export→Test" items.

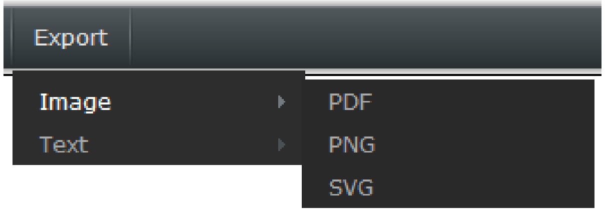
Export
Image
Text
PDF
PNG
SVG
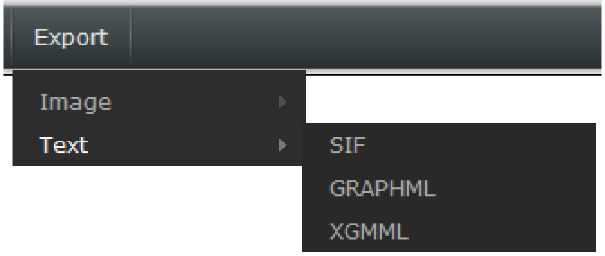
Export
Image
Text
SIF
GRAPHML
XGMML

Appendix A

The background knowledge integrated in KUPNetViz includes the aggregation of several databases for protein-protein interactions, GO terms, KEGG pathways, miRNA to gene interactions etc. Briefly, gene, protein and miRNA annotations are derived from NCBI gene, UniProt and Ensembl, and Microcosm respectively. For the mappings among the various biological entities we used the mapping files provided by NCBI (ftp.ncbi.nlm.nih.gov) as well as the Biomart web services (http://www.biomart.org). Files provided by NCBI were also used for the mapping of gene to their respective GO terms. Molecule interactions are extracted from the STRING protein-protein interaction database (http://www.string-db.org) and miRNA to gene interactions are extracted from the Microcosm miRNA target files (http://www.ebi.ac.uk/enright-srv/microcosm/htdocs/targets/v5/). Finally, biochemical pathway information was derived from KEGG (http://www.genome.jp/kegg/) using its freely available web service Perl API to download and construct mappings between genes and their respective pathways.
